# A microtubule stability switch alters isolated vascular smooth muscle Ca^2+^ flux in response to matrix rigidity

**DOI:** 10.1242/jcs.262310

**Published:** 2024-11-12

**Authors:** Robert T. Johnson, Finn Wostear, Reesha Solanki, Oliver Steward, Alice Bradford, Christopher Morris, Stefan Bidula, Derek T. Warren

**Affiliations:** ^1^ School of Chemistry, Pharmacy and Pharmacology, University of East Anglia, Norwich Research Park, Norwich NR4 7TJ, UK; ^2^ Biomedical Research Centre, University of East Anglia, Norwich Research Park, Norwich NR4 7TJ, Norfolk, UK; ^3^ School of Pharmacy, University College London, London WC1N 1AX, UK

**Keywords:** Ca^2+^ flux, Microtubule, Smooth muscle cell, Matrix rigidity

## Abstract

During ageing, the extracellular matrix of the aortic wall becomes more rigid. In response, vascular smooth muscle cells (VSMCs) generate enhanced contractile forces. Our previous findings demonstrate that VSMC volume is enhanced in response to increased matrix rigidity, but our understanding of the mechanisms regulating this process remain incomplete. In this study, we show that microtubule stability in VSMCs is reduced in response to enhanced matrix rigidity via Piezo1-mediated Ca^2+^ influx. Moreover, VSMC volume and Ca^2+^ flux is regulated by microtubule dynamics; microtubule-stabilising agents reduced both VSMC volume and Ca^2+^ flux on rigid hydrogels, whereas microtubule-destabilising agents increased VSMC volume and Ca^2+^ flux on pliable hydrogels. Finally, we show that disruption of the microtubule deacetylase HDAC6 uncoupled these processes and increased α-tubulin acetylation on K40, VSMC volume and Ca^2+^ flux on pliable hydrogels, but did not alter VSMC microtubule stability. These findings uncover a microtubule stability switch that controls VSMC volume by regulating Ca^2+^ flux. Taken together, these data demonstrate that manipulation of microtubule stability can modify VSMC response to matrix stiffness.

## Teaser

**Highlighted Article:** A mechanistic connection between microtubule stability, Ca^2+^ flux and cell morphology determines the cellular response to matrix rigidity.

## INTRODUCTION

Maintaining aortic compliance, the ability of the aorta to change shape in response to changes in blood pressure is essential for cardiovascular (CV) health. Decreased aortic compliance is a major risk factor associated with a variety of age-related CV diseases (Glasser et al., 1997; Lacolley et al., 2020; Mitchell et al., 2010). The rigidity of the aortic wall is a major contributor to aortic compliance. In the healthy aortic wall, rigidity and compliance are determined by the balance between elastic fibres, including elastin, that provide pliability, and non-elastic fibres, including collagen I, that provide tensile strength to the extracellular matrix (ECM) (Tsamis et al., 2013). However, during ageing and CV disease, elastic fibres degrade and collagen I accumulates. These ECM changes increase the rigidity of the aortic wall and decrease aortic compliance (Afewerki et al., 2019; Ahmed and Warren, 2018; Johnson et al., 2021).

Vascular smooth muscle cells (VSMCs) are the predominant cell type within the aortic wall. VSMC contraction regulates vascular tone in healthy aortae, where aortic wall rigidity and compliance are a balance between ECM rigidity and VSMC stiffness (Johnson et al., 2021). Changes in intracellular calcium ion (Ca^2+^) levels are integral for regulating VSMC contractile function (Ahmed and Warren, 2018). As such, mechanisms that promote release and reuptake of Ca^2+^ from intracellular stores, such as the sarcoplasmic reticulum, are normally tightly regulated. During VSMC contraction, Ca^2+^ release is stimulated via receptor signalling or stretch-activated ion channels, which include the TRPC family and Piezo1, leading to the activation of sarcoplasmic inositol triphosphate receptor (IP_3_R) and ryanodine receptors (Sanders, 2001). This results in a rapid Ca^2+^ spark in VSMCs that drives actomyosin force generation. These sparks are short lived as Ca^2+^ is rapidly reabsorbed to the sarcoplasmic reticulum via SERCA channels (Sanders, 2001).

However, the balance between ECM rigidity and VSMC stiffness is disrupted in ageing and CV disease, resulting in VSMC dysfunction (Lacolley et al., 2020; Tsamis et al., 2013). Age-associated changes in ECM composition increase aortic wall rigidity and trigger increased VSMC stiffness, which further decrease aortic compliance (Lacolley et al., 2020; Sehgel et al., 2015, 2013; Tsamis et al., 2013). This is termed VSMC stiffness syndrome, but our understanding of the mechanisms driving this process remain poorly defined. In response to hypertension, VSMCs undergo a process known as hypertrophy and increase their cell mass without increasing cell number (Owens and Schwartz, 1983; Rizzoni et al., 2000; Schiffrin, 2012; Zhang et al., 2005). Increases in volume and protein synthesis are key components in VSMC hypertrophy, which is known to increase aortic wall thickness and rigidity and decrease aortic compliance (Owens and Schwartz, 1983; Rizzoni et al., 2000; Schiffrin, 2012; Sehgel et al., 2013; Zhang et al., 2005). Importantly, we have recently shown that activation of Piezo1 drives a sustained Ca^2+^ influx, resulting in an increase in VSMC volume, via the membrane translocation of the water channel aquaporin-1, in response to enhanced matrix rigidity (Johnson et al., 2024). Increased matrix rigidity and hypertension are known to promote VSMC dedifferentiation, where they switch from a contractile phenotype to disease-associated synthetic phenotypes (Cao et al., 2022; Hayashi and Naiki, 2009; Xie et al., 2018). Both aquaporin-1 and Piezo1 gene expression is enhanced during VSMC dedifferentiation in atherosclerosis, during carotid remodelling and during aortic ageing (Johnson et al., 2024; Luu et al., 2023). Whether increased volume drives increased VSMC stiffness in response to matrix rigidity and the VSMC phenotypes involved remain unknown.

Microtubules are hollow tube-like structures, made up of α- and β-tubulin heterodimers, that play important roles in determining cell morphology and resisting deformational forces applied to cells (Brangwynne et al., 2006; Stamenović, 2005). Microtubule organisation is regulated via a process called dynamic instability, in which microtubules exist in a balance between growth and shrinkage, which enables them to respond rapidly to the changing needs of a cell (Michaels et al., 2020). Post-translational modifications of tubulin are predicted to regulate microtubule mechanical properties (Song and Brady, 2015). Most modifications are predicted to occur on the outer surface of polymerised microtubules. However, lysine 40 (K40) of α-tubulin, found in the inner lumen of polymerised microtubules, can be acetylated and deacetylated by αTAT1 and HDAC6, respectively (Osseni et al., 2020; Shida et al., 2010). Although the precise impact of K40 acetylation on microtubule mechanical properties remains controversial and contradictory, K40 acetylation is proposed to influence lateral associations between polymerised tubulin monomers and potentially stabilise microtubules, making them more resistant to mechanical damage than deacetylated microtubules (Eshun-Wilson et al., 2019; Janke and Montagnac, 2017; Xu et al., 2017). Enhanced K40 acetylation increases cytoskeletal stiffness in striated muscle, but the impact of K40 acetylation in VSMC matrix rigidity sensing remains unknown (Coleman et al., 2021).

The mechanical regulation of VSMC behaviour obeys well-defined rules. For example, in response to enhanced matrix rigidity, VSMCs generate increased actomyosin-derived traction forces and increased deformational stresses are placed upon the cell membrane (Petit et al., 2019; Sanyour et al., 2019). These deformational stresses drive the opening of stretch-activated ion channels in VSMCs (Johnson et al., 2024). Microtubules exist in a mechanical balance with actomyosin activity, serving as pre-stressed compression-bearing struts capable of resisting actomyosin-generated deformational stresses (Brangwynne et al., 2006; Johnson et al., 2021; Stamenović, 2005). This relationship is known as the tensegrity model (Stamenović, 2005). The tensegrity model predicts the interplay between actomyosin and microtubules in VSMCs. Previous studies have shown that microtubule stabilisation decreases VSMC actomyosin activity, whereas microtubule destabilisation increases VSMC actomyosin activity (Ahmed et al., 2022; Zhang et al., 2000). Other models have been proposed that describe VSMC mechano-adaption responses, yet we lack an in-depth mechanistic understanding of pathways linking changes in VSMC behaviour to the proposed models of mechanotransduction (Steucke et al., 2017). For example, whether mechanical cues and stretch-activated ion channel activity influence microtubule stability and vice versa remains unknown.

In the current study, we used pliable and rigid polyacrylamide hydrogels to model healthy and diseased/aged aortic wall stiffness (Ahmed et al., 2022; Johnson et al., 2024). We show that changes in microtubule stability serve as a switch that regulates Ca^2+^ flux and VSMC volume control. Treatment with the microtubule-stabilising agent paclitaxel reduced VSMC volume on rigid hydrogels. In contrast, treatment with the microtubule-destabilising agent colchicine increased VSMC volume on pliable hydrogels. Furthermore, we show that paclitaxel diminished Ca^2+^ flux specifically in VSMCs on rigid hydrogels, whereas colchicine treatment prolonged Ca^2+^ flux in VSMCs on pliable hydrogels. In keeping with our previous findings, our data also implicate Piezo1 in this process, and depletion of Piezo1 increased VSMC microtubule stability specifically on rigid hydrogels. Finally, we demonstrate that disruption of HDAC6 increases acetylation of α-tubulin and induces increased volume and Ca^2+^ flux in VSMCs on pliable hydrogels. Taken together, these data implicate a Piezo1–Ca^2+^–microtubule stability feedback pathway in the regulation of VSMC matrix rigidity response.

## RESULTS

### Piezo1 activity promotes microtubule destabilisation within VSMCs on rigid hydrogels

**Fig. 1.**
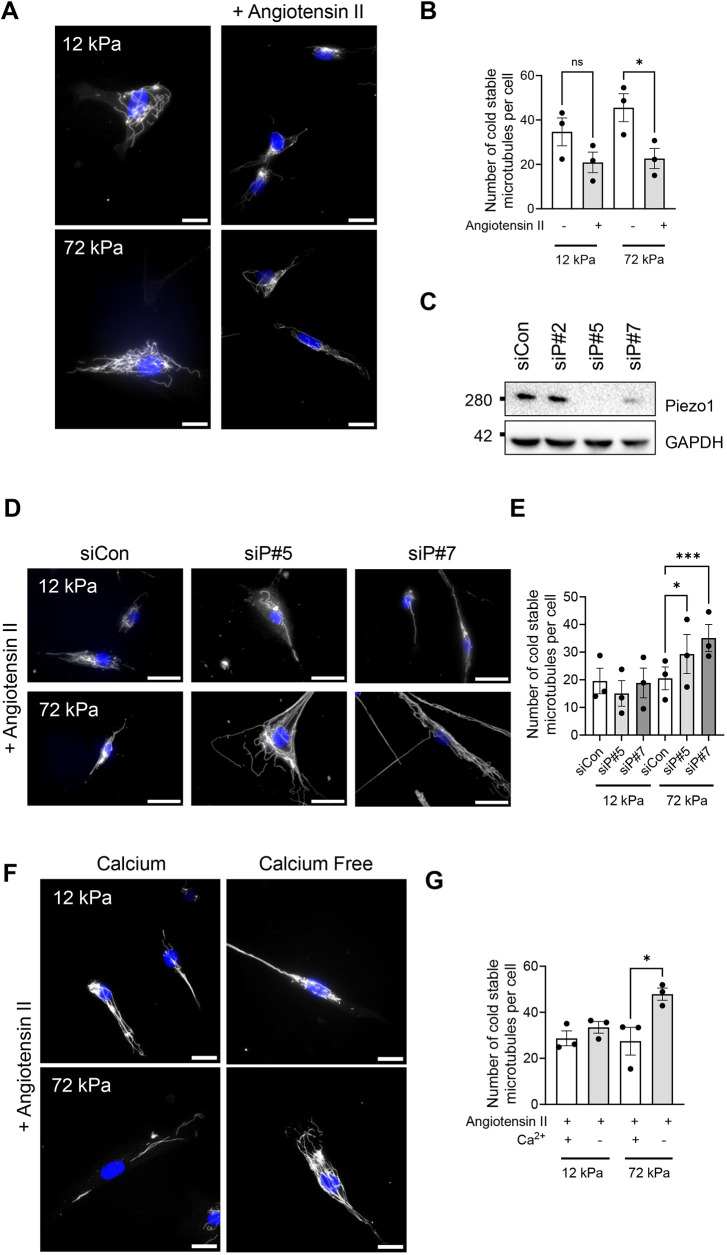
**Extracellular Ca^2+^ influx via Piezo1 ion channels triggers microtubule destabilisation.** (A) Representative images of isolated vascular smooth muscle cells (VSMCs) cultured on 12 or 72 kPa polyacrylamide hydrogels with or without angiotensin II treatment. Cold-stable microtubules (α-tubulin, grey) and nuclei (DAPI, blue) are shown. Scale bars: 50 μm. (B) The graph shows the number of cold-stable microtubules per cell for 12 kPa and 72 kPa hydrogel cultures with or without angiotensin II treatment. The graph represents combined data from three independent experiments, analysing ≥56 cells per condition, and black dots mark mean data for each independent repeat. (C) Representative western blots confirming efficient siRNA-mediated Piezo1 depletion in VSMCs (siCon, non-targeting scrambled siRNA; siP#5 and siP#7, independent Piezo1-targeting siRNAs #5 and #7). (D) Representative images of angiotensin II-stimulated siCon-, siP#5- and siP#7-treated VSMCs cultured on 12 or 72 kPa polyacrylamide hydrogels. Cold-stable microtubules, (α-tubulin, grey) and nuclei (DAPI, blue) are shown. Scale bars: 50 μm. (E) The graph shows the number of cold-stable microtubules per cell on 12 and 72 kPa hydrogels in angiotensin II-stimulated siCon-, siP#5- and siP#7-treated VSMCs. The graph represents combined data from three independent experiments, analysing ≥65 cells per condition, and black dots mark mean data for each independent repeat. (F) Representative images of angiotensin II-stimulated VSMCs cultured on 12 or 72 kPa polyacrylamide hydrogels in Ca^2+^-containing or Ca^2+^-free medium. Cold-stable microtubules (α-tubulin, grey) and nuclei (DAPI, blue) are shown. Scale bars: 50 μm. (G) The graph shows the number of cold-stable microtubules per cell on 12 and 72 kPa hydrogels in angiotensin II-stimulated VSMCs in the presence or absence of extracellular Ca^2+^. The graph represents combined data from three independent experiments, analysing ≥71 cells per condition, and black dots mark mean data for each independent experimental repeat. Significance was determined using a two-way ANOVA followed by Tukey's post hoc test. ns, not significant; *P<0.05; ***P<0.001. Error bars represent ±s.e.m.

To investigate the response of VSMCs to matrix rigidity, cells were seeded onto polyacrylamide hydrogels that mimic healthy aortae (12 kPa pliable hydrogels) or diseased/aged aortae (72 kPa rigid hydrogels) (Ahmed et al., 2022; Johnson et al., 2024). Previous studies have shown that matrix rigidity promotes increased VSMC traction stress generation. To confirm that angiotensin II-stimulated VSMCs seeded on rigid hydrogels generated enhanced traction stresses, we performed traction force microscopy. As expected, analysis confirmed that angiotensin II-stimulated VSMCs seeded on rigid hydrogels generated greater maximal and total traction stress compared to that generated by their counterparts on pliable hydrogels (Fig. S1). Our previous study found that on pliable hydrogels, VSMCs generated enhanced traction stresses as a result of colchicine-induced microtubule depolymerisation. Therefore, we predicted that VSMCs would display decreased microtubule stability on rigid hydrogels. To test this, we compared the number of cold-stable microtubules in quiescent VSMCs and by angiotensin II-stimulated VSMCs on pliable and rigid hydrogels. Analysis revealed that angiotensin II stimulation resulted in a reduction of cold-stable microtubules in VSMCs on pliable and rigid hydrogels (Fig. 1A,B). However, this decrease was only significantly different on rigid hydrogels (Fig. 1A,B).

We have previously shown that Piezo1 is an essential mediator of the enhanced VSMC volume response on rigid hydrogels. We next used a siRNA-mediated knockdown approach to investigate whether Piezo1 was also required for this change in microtubule stability. Consistent with our previous studies, western blot analysis confirmed that Piezo1 was efficiently depleted in VSMCs by Piezo1-specific siRNA compared to non-targeting scrambled siRNA (Fig. 1C). Piezo1 depletion had no effect on VSMC viability (Fig. S1D). Analysis of microtubule stability revealed that Piezo1-depleted, angiotensin II-stimulated VSMCs possessed similar numbers of cold-stable microtubules on pliable hydrogels as their scrambled control siRNA-treated counterparts (Fig. 1D,E). In contrast, Piezo1-depleted, angiotensin II stimulated VSMCs on rigid hydrogels possessed increased numbers of cold-stable microtubules compared to their scrambled control siRNA-treated counterparts (Fig. 1D,E). We have previously shown that Piezo1 activation promotes Ca^2+^ influx in VSMCs on rigid hydrogels. To confirm that Ca^2+^ influx was driving microtubule destabilisation, we performed a cold-stable microtubule assay in the presence or absence of extracellular Ca^2+^. The absence of extracellular Ca^2+^ had no effect on the number of cold-stable microtubules detected in angiotensin II-stimulated VSMCs on pliable hydrogels (Fig. 1F,G). In contrast, the number of cold-stable microtubules increased within angiotensin II-stimulated VSMCs on rigid hydrogels when extracellular Ca^2+^ was absent (Fig. 1F,G). This suggests that Piezo1-mediated Ca^2+^ influx decreases microtubule stability on rigid hydrogels following angiotensin II stimulation.

### Microtubule stability influences isolated smooth muscle cell volume following contractile agonist stimulation

**Fig. 2.**
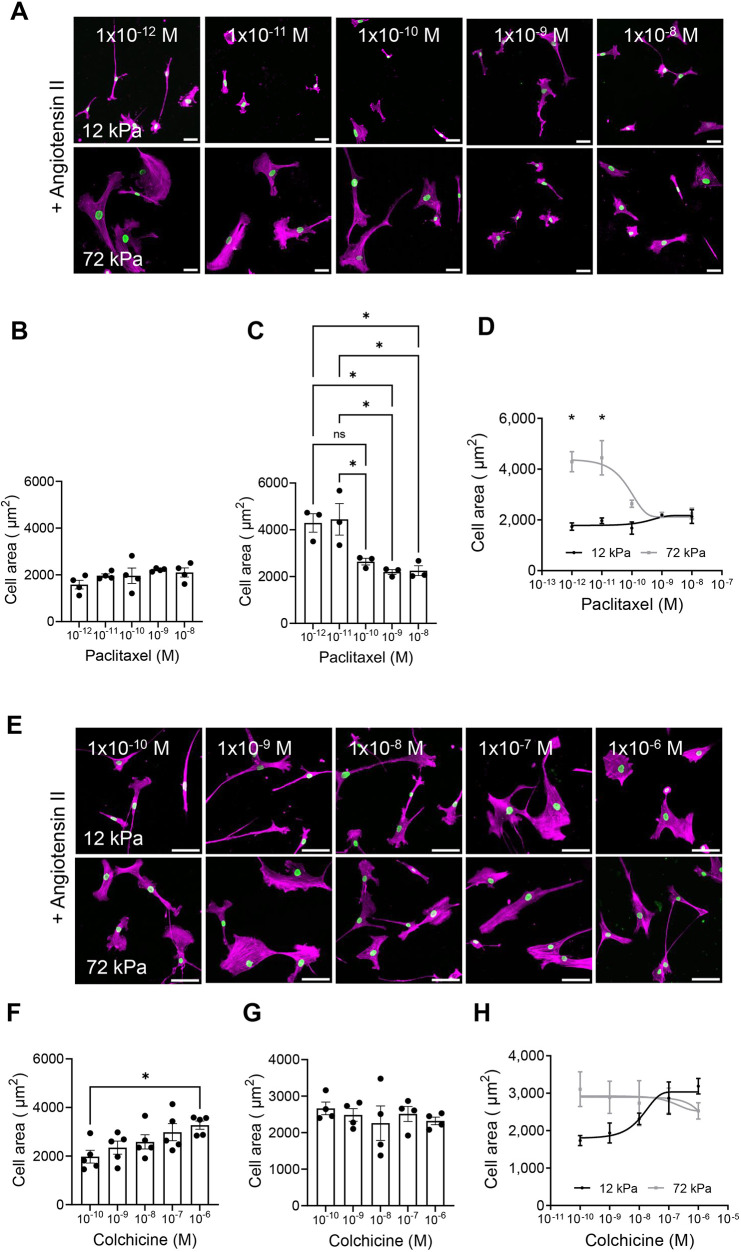
**Microtubule stability influences VSMC spreading following angiotensin II stimulation.** (A) Representative images of isolated VSMCs cultured on 12 or 72 kPa polyacrylamide hydrogels pre-treated with a concentration range of paclitaxel prior to angiotensin II stimulation. The actin cytoskeleton (purple) and lamin A/C-labelled nuclei (green) are shown. Scale bars: 50 μm. (B–D) The graphs show VSMC area on (B) 12 kPa hydrogels, (C) 72 kPa hydrogels, and (D) 12 and 72 kPa hydrogels. Graphs represent the combined data from three or four independent experiments, analysing ≥80 cells per condition. Black dots represent mean data from each individual experimental repeat. (E) Representative images of isolated VSMCs cultured on 12 or 72 kPa polyacrylamide hydrogels pre-treated with a concentration range of colchicine prior to angiotensin II stimulation. The actin cytoskeleton (purple) and lamin A/C-labelled nuclei (green) are shown. Scale bars: 50 μm. (F–H) The graphs show VSMC area on (F) 12 kPa hydrogels, (G) 72 kPa hydrogels, and (H) 12 and 72 kPa hydrogels. Graphs represent the combined data from four or five independent experiments, analysing ≥80 cells per condition. Black dots represent mean data from each individual experimental repeat. Significance was determined using one-way ANOVA (B,C,F,G) or two-way ANOVA (D,H) followed by Tukey's post hoc test. ns, not significant; *P<0.05. Error bars represent ±s.e.m.

The above data show that, on rigid substrates, VSMCs generate increased traction stresses and possess decreased microtubule stability. We have previously shown that VSMCs on rigid hydrogels swell and possess increased cell area and volume. We next hypothesised that decreased microtubule stability was driving the enhanced VSMC volume response on rigid hydrogels. To test this, we utilised our screening assay described previously (Ahmed et al., 2022; Johnson et al., 2024). Quiescent VSMCs were seeded onto pliable and rigid hydrogels and pre-treated with increasing concentrations of microtubule stabilisers prior to angiotensin II stimulation. Treatment with either paclitaxel or epothilone B had no effect on the contractile response of VSMCs seeded on pliable hydrogels (Fig. 2A,B,D; Fig. S2). In contrast, increasing concentrations of either microtubule stabiliser was sufficient to prevent the increase in VSMC area observed following angiotensin II stimulation on rigid hydrogels (Fig. 2A,C,D; Fig. S2). Given that microtubule stabilisation prevented VSMC enlargement following angiotensin II stimulation on rigid hydrogels, we next hypothesised that microtubule destabilisation would trigger VSMC enlargement in angiotensin II-stimulated VSMCs on pliable hydrogels. VSMCs were pre-treated with increasing concentrations of the microtubule destabilisers colchicine or nocodazole, and then stimulated with angiotensin II. On pliable hydrogels, VSMCs treated with either colchicine or nocodazole displayed increased cell area following angiotensin II stimulation (Fig. 2E,F,H; Fig. S3). In contrast, treatment with the microtubule destabilisers had no effect on VSMCs seeded on rigid hydrogels (Fig. 2E,G,H; Fig. S3). All microtubule targeting agents were used at concentrations that did not cause cell death, as confirmed through a viability assay for concentrations of epothilone B and nocodazole (Fig. S4A,B) or previously shown for paclitaxel and colchicine (Ahmed et al., 2022). We next confirmed that paclitaxel and colchicine treatments were inducing microtubule stabilisation and destabilisation, respectively. Quiescent VSMCs seeded on pliable and rigid hydrogels were pre-treated with microtubule-targeting agents prior to angiotensin II stimulation. Analysis confirmed that paclitaxel treatment (1 nM) resulted in increased numbers of cold-stable microtubules in angiotensin II-stimulated VSMCs on both rigid and pliable hydrogels (Fig. S4C,D). Colchicine treatment (100 nM) resulted in reduced numbers of cold-stable microtubules in angiotensin II-stimulated VSMCs on pliable hydrogels but did not significantly alter the number of cold-stable microtubules in angiotensin II-treated VSMCs on rigid hydrogels (Fig. S4E,F).

**Fig. 3.**
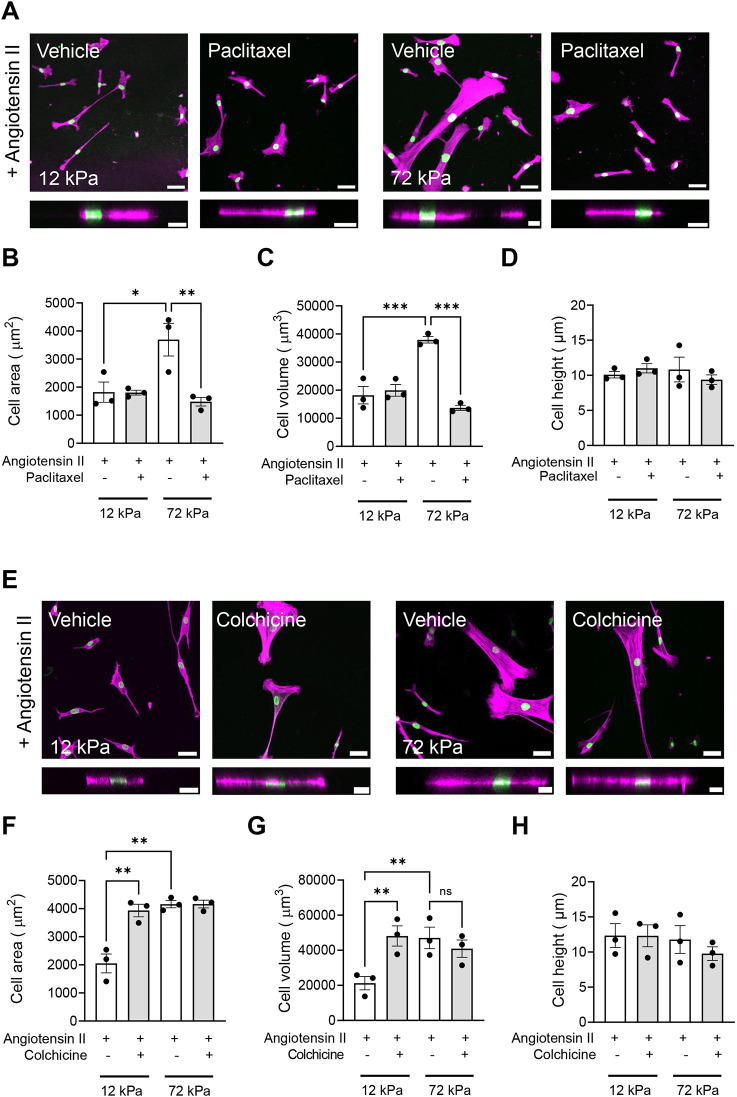
**VSMC volume is regulated by microtubule stability.** (A) Representative images of isolated VSMCs cultured on 12 or 72 kPa polyacrylamide hydrogels pre-treated with vehicle control (dH_2_O) or paclitaxel prior to angiotensin II stimulation. The actin cytoskeleton (purple) and DAPI-labelled nuclei (green) are shown. Top panels show representative *xy* images of VSMC area, and bottom panels show representative *xz* images of VSMC height. Scale bars: 50 μm (top); 20 μm (bottom). (B–D) Graphs show VSMC (B) area, (C) volume and (D) height and represent combined data from three independent experiments, with ≥45 cells analysed per condition. Mean data from each individual repeat of the three independent experiments are shown by black dots. (E) Representative images of isolated VSMCs cultured on 12 or 72 kPa polyacrylamide hydrogels pre-treated with vehicle control (dH_2_O) or colchicine prior to angiotensin II stimulation. The actin cytoskeleton (purple) and DAPI-labelled nuclei (green) are shown. Top panels show representative *xy* images of VSMC area, and bottom panels show representative *xz* images of VSMC height. Scale bars: 50 μm (top); 20 μm (bottom). (F–H) Graphs show VSMC (F) area, (G) volume and (H) height and represent the combined data from three independent experiments, with ≥42 cells analysed per condition. Mean data from each individual repeat of the three independent experiments are shown by black dots. Significance was determined using two-way ANOVA followed by Tukey's test. ns, not significant; *P<0.05; **P<0.01; ***P<0.001. Error bars represent±s.e.m.

Having determined that microtubule stability regulated changes in VSMC area, we then sought to confirm its regulation of VSMC volume. Quiescent VSMCs were pre-treated with paclitaxel (1 nM) or colchicine (100 nM) prior to angiotensin II stimulation and confocal microscopy was used to assess changes in cell volume. Treatment with the microtubule stabiliser paclitaxel had no effect on the volume of VSMCs seeded on pliable hydrogels (Fig. 3A–D), whereas, on rigid hydrogels, microtubule stabilisation prevented the angiotensin II-induced expansion of VSMC volume (Fig. 3A–D). Finally, microtubule destabilisation via colchicine pre-treatment increased VSMC volume on pliable hydrogels following angiotensin II stimulation (Fig. 3E–H) but had no additional effect on VSMC volume on rigid hydrogels (Fig. 3E–H).

### Changes in microtubule stability alter Ca^2+^ flux

**Fig. 4.**
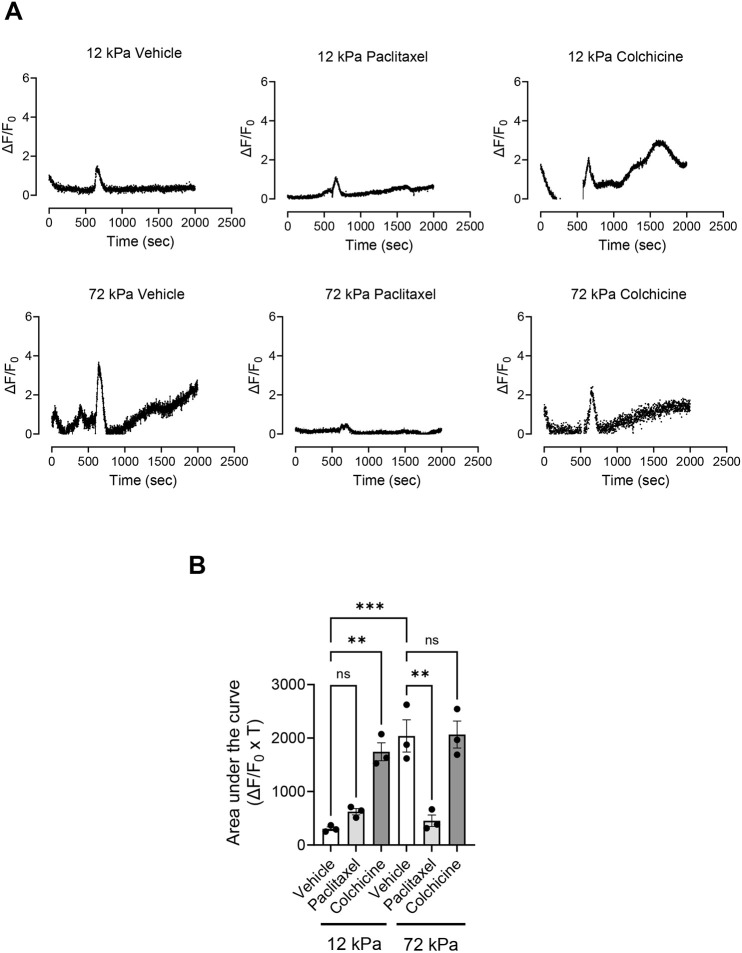
**Microtubule stability influences Ca^2+^ flux in VSMCs.** (A) Graphs show representative ΔF/F_0_ values over time for vehicle control-, paclitaxel- and colchicine-pre-treated, angiotensin II-stimulated, Fluo-4-loaded VSMCs on 12 and 72 kPa hydrogels, where F represents the fluorescence intensity. Each graph represents the mean combined data of nine individual fields of view combined from three individual experiments. (B) The graph shows area under the curve values (ΔF/F_0_×T, where T is time). Mean data for individual repeats of the three independent experiments are shown as black dots. Significance was determined using two-way ANOVA followed by Tukey's multiple comparison test. ns, not significant; **P<0.01; ***P<0.001. Error bars represent ±s.e.m.

The above data confirm that microtubule stability influences VSMC volume control. Previously, we identified Piezo1-mediated Ca^2+^ influx as a regulator of VSMC volume control. Therefore, we next hypothesised that microtubule stability was altering Ca^2+^ flux in VSMCs. To test this, quiescent VSMCs on rigid and pliable hydrogels were loaded with the Ca^2+^ indicator Fluo-4 prior to angiotensin II treatment. Fluorescence video time-lapse microscopy was used to measure changes in Fluo-4 fluorescence. Analysis supported our previous findings showing that Ca^2+^ flux was heightened and prolonged in VSMCs on rigid hydrogels compared to that in VSMCs on pliable hydrogels (Fig. 4A,B). Colchicine treatment resulted in prolonged Ca^2+^ flux in VSMCs on pliable hydrogels but had little effect on Ca^2+^ flux in VSMCs on rigid hydrogels (Fig. 4A,B). In contrast, paclitaxel treatment reduced the Ca^2+^ flux in VSMCs on rigid hydrogels but had no effect on Ca^2+^ flux in VSMCs on pliable hydrogels (Fig. 4A,B).

### HDAC6 disruption increases microtubule acetylation and induces enhanced VSMC volume response on pliable hydrogels

**Fig. 5.**
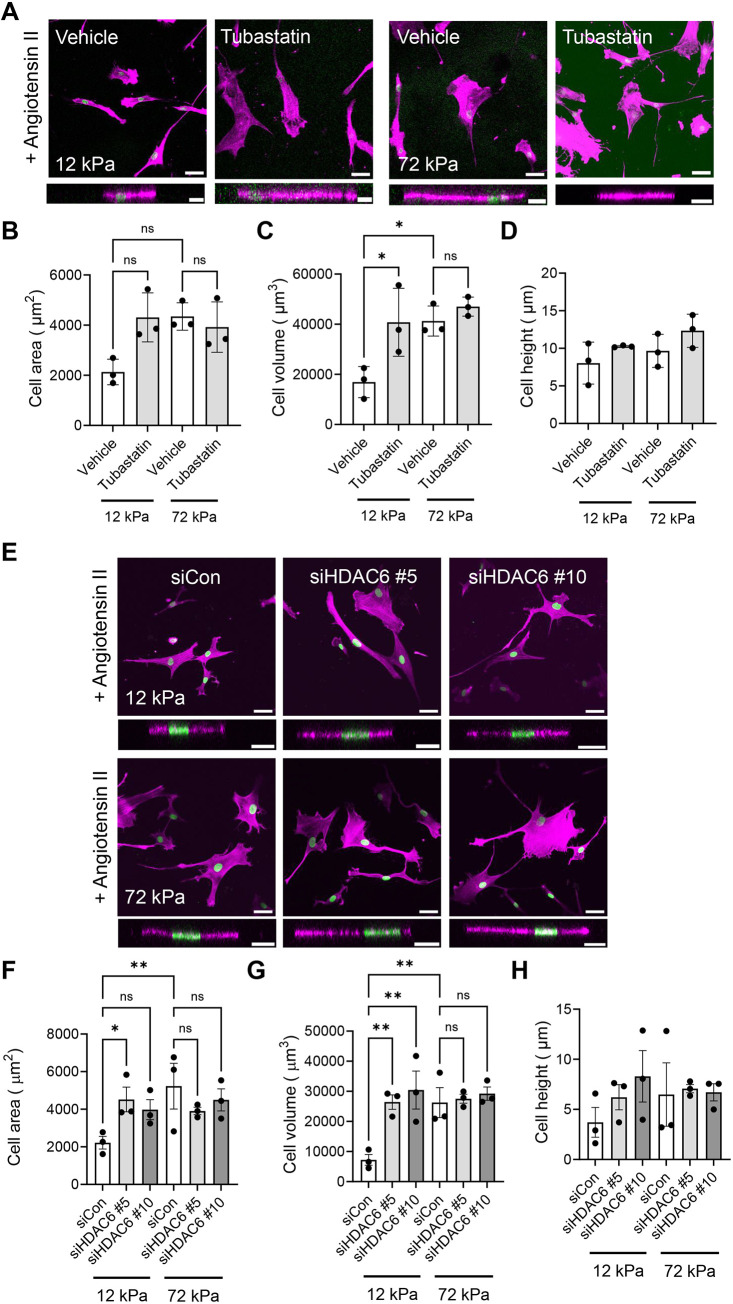
**HDAC6 disruption enhances VSMC volume.** (A) Representative images of isolated VSMCs cultured on 12 or 72 kPa polyacrylamide hydrogels pre-treated with vehicle control (DMSO) or tubastatin prior to angiotensin II stimulation. The actin cytoskeleton (purple) and DAPI-labelled nuclei (green) are shown. Top panels show representative *xy* images of VSMC area, and bottom panels show representative *xz* images of VSMC height. Scale bars: 50 μm (top); 20 μm (bottom). (B–D) Graphs show VSMC (B) area, (C) volume and (D) height and represent the combined data from three independent experiments with ≥41 cells analysed per condition. Mean data from each individual repeat of the three independent experiments are shown by black dots. (E) Representative images of isolated VSMCs cultured on 12 or 72 kPa polyacrylamide hydrogels treated with scrambled (siCon) or HDAC6-targeting (siHDAC6 #5 and #10) siRNA oligonucleotides prior to angiotensin II stimulation. The actin cytoskeleton (purple) and DAPI-labelled nuclei (green) are shown. Top panels show representative *xy* images of VSMC area, and bottom panels show representative *xz* images of VSMC height. Scale bars: 50 μm (top); 20 μm (bottom). (F–H) Graphs show VSMC (F) area, (G) volume and (H) height and represent the combined data from three independent experiments, with ≥30 cells analysed per condition. Mean data from each individual repeat of the three independent experiments are shown by black dots. Significance was determined using two-way ANOVA followed by Tukey's test. ns, not significant; *P<0.05; **P<0.01. Error bars represent ±s.e.m.

**Fig. 6.**
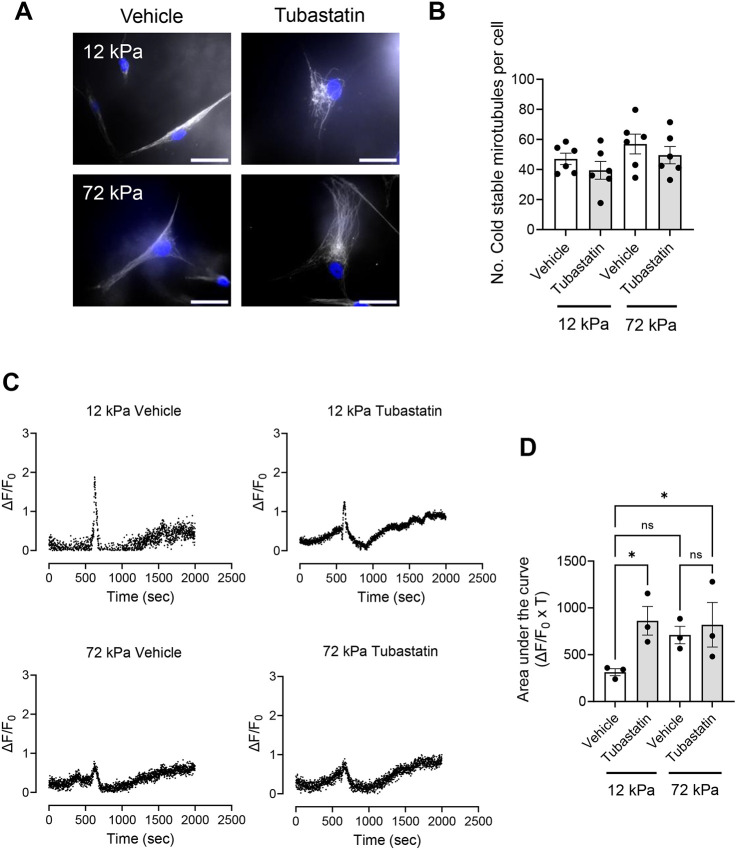
**Tubastatin treatment alters Ca^2+^ flux in VSMCs.** (A) Representative images of isolated angiotensin II-stimulated VSMCs pre-treated with vehicle control or tubastatin on 12 or 72 kPa polyacrylamide hydrogels. Cold-stable microtubules (α-tubulin, grey) and nuclei (DAPI, blue) are shown. Scale bars: 50 μm. (B) The graph shows the number of cold-stable microtubules per cell on 12 and 72 kPa hydrogels. The graph represents combined data from six independent experiments, analysing >60 cells per condition, and black dots mark mean data for each independent repeat. (C) Graphs show representative ΔF/F_0_ values over time for vehicle control and tubastatin-pre-treated, angiotensin II-stimulated, Fluo-4-loaded VSMCs on 12 and 72 kPa hydrogels. Each graph represents the mean combined data of nine individual fields of view combined from three individual experiments. (D) The graph shows area under the curve values (ΔF/F_0_×T). Mean data for individual repeats of the three independent experiments are shown as black dots. Significance was determined using two-way ANOVA followed by Tukey's multiple comparison test. ns, not significant; *P<0.05. Error bars represent±s.e.m.

Although controversial, α-tubulin K40 acetylation is proposed to alter the mechanical properties of microtubules. We next hypothesised that increased tubulin acetylation would influence the VSMC matrix rigidity response. To test this, we used tubastatin to inhibit HDAC6 deacetylation of the K40 position. Western blot analysis confirmed that VSMCs exposed to an increasing concentration of tubastatin possessed increased levels of acetylated α-tubulin (Fig. S5A,B). Next, quiescent VSMCs were pre-treated with tubastatin (1 µM) prior to angiotensin II stimulation and confocal microscopy was used to assess changes in the cell volume. Tubastatin-treated VSMCs possessed increased volume compared to their vehicle-treated counterparts on pliable hydrogels (Fig. 5A–D). Tubastatin treatment had no effect on VSMC volume on rigid hydrogels (Fig. 5A–D). To confirm that this altered response was driven by HDAC6 disruption, we performed siRNA-mediated HDAC6 depletion experiments. WB confirmed HDAC6 was efficiently depleted in VSMCs by two independent siRNA oligomers (Fig. S5C,D). HDAC6 depletion had no effect on VSMC viability (Fig. S5E). Analysis of *z*-stacks captured by confocal microscopy confirmed that HDAC6-depleted, angiotensin II-stimulated VSMCs possessed increased volume on pliable hydrogels compared to that of their scrambled control siRNA-treated counterparts (Fig. 5E–H). HDAC6 knockdown had no effect on VSMC volume on rigid hydrogels (Fig. 5E–H). Owing to the increased volume of HDAC6-disrupted VSMCs on pliable hydrogels, we next predicted that increased tubulin acetylation was reducing VSMC microtubule stability and prolonging Ca^2+^ flux. To test these possibilities, we firstly performed cold-stable microtubule stability assays in tubastatin-treated VSMCs on pliable and rigid hydrogels. However, the number of cold-stable microtubules remained unaffected by tubastatin treatment (Fig. 6A,B). Secondly, quiescent VSMCs on rigid and pliable hydrogels were loaded with the Ca^2+^ indicator Fluo-4 prior to angiotensin II treatment. Fluorescence video time-lapse microscopy was used to measure changes in Fluo-4 fluorescence. Tubastatin treatment resulted in prolonged Ca^2+^ flux in VSMCs on pliable hydrogels but had little effect on Ca^2+^ flux in VSMCs on rigid hydrogels (Fig. 6C,D). Finally, we investigated whether matrix rigidity influenced α-tubulin K40 acetylation levels. However, western blotting analysis revealed that K40 acetylation levels were similar in the lysates of VSMCs grown on pliable and rigid hydrogels (Fig. S6A,B). Finally, we performed western blotting to determine whether Piezo1 and HDAC6 levels are influenced by matrix rigidity. As previously reported, Piezo1 levels appeared to decrease in VSMCs on rigid hydrogels compared to those in VSMCs on pliable hydrogels (Johnson et al., 2024). HDAC6 levels remained similar in VSMCs on pliable and rigid hydrogels (Fig. S6C).

## DISCUSSION

Despite much research, our understanding of the mechanisms driving VSMC dysfunction and its contribution to decreased aortic compliance in ageing and CV disease remains limited. Previous studies have shown that enhanced matrix stiffness promotes the dedifferentiation of VSMCs, downregulating contractile markers while increasing the expression of proliferative genes (Brown et al., 2010; Nagayama and Nishimiya, 2020; Sazonova et al., 2015; Xie et al., 2018). Increased VSMC migrational capacity, adhesion, proliferation and volume have also been reported (Brown et al., 2010; Krajnik et al., 2023; Nagayama and Nishimiya, 2020; Rickel et al., 2020; Sazonova et al., 2015; Wong et al., 2003). Furthermore, in response to matrix stiffness, VSMCs reorganise their actin cytoskeleton and generate enhanced traction stresses, a finding we recapitulate in this study (Brown et al., 2010; Petit et al., 2019; Sanyour et al., 2019; Sazonova et al., 2015).

Our findings show that changes in the tensegrity equilibrium defines VSMC behaviour in response to matrix rigidity. In this model, the microtubules serve as compression-bearing struts that resist actomyosin-generated strain (Brangwynne et al., 2006; Stamenović, 2005). Healthy VSMC behaviour is therefore a balance between microtubule stability and actomyosin activity. Actomyosin-generated tension drives VSMC contraction and microtubules maintain VSMC morphology, protecting against strain-induced cellular damage (Stamenović, 2005). The tensegrity model predicts that disruption of the microtubule cytoskeleton alters the equilibrium of these stresses and results in increased VSMC traction stress generation, a hypothesis previously confirmed by both wire myography and traction force microscopy (Ahmed et al., 2022; Zhang et al., 2000). We show that in rigid environments, the VSMC tensegrity equilibrium becomes unbalanced. Contractile agonist stimulation of VSMCs on rigid substrates increases traction stress generation and reduces the number of cold-stable microtubules, resulting in deregulation of VSMC morphology and enlargement of VSMC volume. Importantly, the altered tensegrity equilibrium can be induced by microtubule-destabilising agents on pliable substrates and restored by treatment with agents that stabilise microtubules on rigid substrates. These data suggest that microtubules and the tensegrity equilibrium are key regulators of VSMC function and dysfunction. Given the prevalence of increased aortic wall stiffness in ageing and disease, our findings suggest that altered tensegrity equilibrium is a key driver of VSMC dysfunction under these conditions (Tsamis et al., 2013). We propose that changing this equilibrium could be a mechanistic target for manipulating VSMC function. Owing to their toxicity, using microtubule-targeting agents clinically is not a viable option to modulate this equilibrium; thus, a better understanding of the molecular mechanisms that regulate this equilibrium is now required to test this idea further.

In this study, we used Ca^2+^ flux as a readout of stretch-activated channel function. One of these channels, Piezo1, is emerging as an important regulator of VSMC dysfunction (Johnson et al., 2024; Luu et al., 2023; Qian et al., 2022; Retailleau et al., 2015; Swiatlowska et al., 2023). Our findings further implicate Piezo1 as a critical regulator of VSMC behaviour and suggest that changes in Piezo1 opening drive altered VSMC behaviour in response to enhanced matrix stiffness. In this study, we show that VSMCs on rigid hydrogels display prolonged Ca^2+^ flux, increased traction stresses and reduced microtubule stability. The relationship between Ca^2+^ flux and microtubule stability were reciprocal, and changes in microtubule stability altered Ca^2+^ flux. We have previously shown that changes in microtubule stability also altered VSMC traction stress generation. Although untested, we predict that microtubule stability alters the ability of VSMCs to withstand the deformational forces placed on the plasma membrane. This would reduce membrane tension and prevent the opening of stretch-activated ion channels. Therefore, stretch-activated ion channel-mediated Ca^2+^ influx serves as a mechanistic switch that triggers destabilisation of microtubule-based compression-bearing struts and shifts the VSMC tensegrity equilibrium. We still do not understand the precise mechanisms that drive Piezo1-mediated microtubule destabilisation. Millimolar Ca^2+^ concentrations spontaneously induce microtubule destabilisation *in vitro*, but cellular concentrations are much lower than this. We envisage three possibilities: (1) prolonged Ca^2+^ flux induces changes in the ability of microtubule-stabilising proteins to associate with microtubules, resulting in destabilisation; (2) increased mechanical damage induces microtubule catastrophe; or (3) increased Ca^2+^ reduces microtubule growth or polymerisation.

The role of acetylation in microtubule stability, rigidity and resistance to mechanical damage remains controversial with many conflicting reports. Our findings suggest that changes in microtubule post-translational modification can influence Ca^2+^ flux and VSMC volume on pliable hydrogels. Despite this finding, K40 acetylation remained unaltered between VSMCs on pliable and rigid hydrogels in our study. This suggests that K40 acetylation does not play a role in the altered microtubule stability observed during the VSMC matrix rigidity response. HDAC6 inhibition also failed to alter microtubule stability and appeared to uncouple the tensegrity equilibrium from the Ca^2+^ flux response on pliable hydrogels. Despite this apparent uncoupling, the increased Ca^2+^ flux activated downstream signalling to enhance VSMC volume following angiotensin II stimulation. HDAC6 has numerous targets, and we predict that this uncoupling is being driven by increased acetylation of other targets that remain unknown. Importantly, these findings suggest that, in addition to the tensegrity equilibrium and our previously identified volume control pathway, other unidentified mechanisms also contribute to the VSMC response to matrix rigidity. Further research is now needed to identify these unknown mechanisms for a more complete understanding of the VSMC matrix rigidity response.

In the healthy aortic wall, VSMCs adopt a quiescent contractile differentiated phenotype. During ageing and disease, VSMCs downregulate contractile marker expression and modulate their phenotype towards disease-relevant phenotypes (Pedroza et al., 2020; Wirka et al., 2019; Worssam et al., 2023). An important limitation of our study is that we used dedifferentiated isolated VSMCs. VSMCs are known to possess multiple stretch-activated ion channels, and these can vary depending on phenotype. We have previously shown that Piezo1 is upregulated in disease-relevant phenotypes (Johnson et al., 2024). It remains unknown whether the VSMC tensegrity equilibrium can be altered in healthy contractile differentiated VSMCs, but microtubule-targeting agents have been shown to alter VSMC contractile function in isolated aortic rings, suggesting that this is the case (Zhang et al., 2000). VSMC hypertrophy is an early indicator of dysfunction. Hypertrophy comprises both an increase in cell volume and an increase in protein synthesis. It remains unknown whether the increased volume response of VSMCs on rigid hydrogels is accompanied by increased protein synthesis. It also remains unknown whether increased VSMC volume contributes to decrease aortic compliance further. Given that water is essentially incompressible under normal conditions, increased VSMC volume is likely to contribute to the pathophysiological aortic wall biomechanics. More research is needed to address these current gaps in our knowledge.

In this study, we implicate Piezo1 activation as a driver of matrix rigidity-induced deregulation of the VSMC tensegrity equilibrium. However, we cannot rule out the possibility that other stretch-activated ion channels can also induce altered tensegrity equilibrium in other VSMC phenotypes. Despite this limitation, our findings support the notion that the VSMC tensegrity equilibrium is modifiable and can be targeted to restore VSMC morphology.

## MATERIALS AND METHODS

### Polyacrylamide hydrogel preparation

Hydrogels were prepared as described previously (Johnson et al., 2024; Minaisah et al., 2016). Briefly, glass coverslips were activated by treating with (3-aminopropyl)triethoxysilane (Merck) for 2 min, washed three times in dH_2_O, then fixed in 0.5% glutaraldehyde for 40 min. After fixation, coverslips were washed and left to air dry overnight. The polyacrylamide hydrogel buffer comprised: 12 kPa – 7.5% acrylamide, 0.15% bis-acrylamide in dH_2_O; 72 kPa – 10% acrylamide, 0.5% bis-acrylamide in dH_2_O. To prepare hydrogels for fabrication, the appropriate volume of buffer was supplemented with 10% ammonium persulphate (APS; 1:100) and tetramethylethylenediamine (TEMED; 1:1000), then placed on a standard microscopy slide and covered with an activated coverslip. Once set, the hydrogels were washed three times in dH_2_O, cross-linked with sulpho-SANPAH (1:3000; Merck) under ultraviolet illumination (365 nm) for 5 min, then functionalised with collagen I (0.1 mg/ml; Thermo Fisher Scientific) for 10 min at room temperature. Hydrogel stiffness was previously confirmed using a JPK Nanowizard-3 atomic force microscope (Porter et al., 2020).

### VSMC culture

Human adult aortic VSMCs (passages 3–10) were purchased from Cell Applications (354-05a). Standard VSMC culture was performed as previously described (Ahmed et al., 2022; Johnson et al., 2024). VSMCs were seeded onto polyacrylamide hydrogels in basal medium (Cell Applications, 310-500) 18 h prior to the beginning of the experiment to induce quiescence. Briefly, VSMCs were pre-treated for 30 min, prior to co-treatment with the contractile agonist angiotensin II (10 µM) for an additional 30 min. Specific experimental concentrations are provided in the corresponding figure legends. For experiments performed in growth media, VSMCs were seeded onto hydrogels and incubated overnight. Please see Table S1 for details of compounds used in this study.

### siRNA knockdown

VSMC siRNA transfection was performed using HiPerFect transfection reagent (Qiagen), as per the manufacturer's instructions, the day before cells were seeded onto hydrogels. VSMCs were transfected with either scrambled siRNA control, Piezo1-targeting siRNA (siRNA #5, 5ʹ-CCGCGTCTTCCTTAGCCATTA-3ʹ; siRNA #7, 5ʹ-CGGCCGCCTCGTGGTCTACAA-3ʹ) or HDAC6 targeting siRNA (siRNA #5, 5ʹ-CACCGTCAACGTGGCATGGAA-3ʹ; siRNA #10, 5ʹ-CCGGAGGGTCCTTATCGTAGA-3ʹ) oligonucleotides. The next afternoon, cells were seeded onto hydrogels as above and serum was withdrawn overnight to induce quiescence, and the next morning, VSMCs were stimulated with angiotensin II (10 µM) for 30 min prior to fixation and downstream immunofluorescence analysis.

### Western blotting

Western blotting was performed as previously described (Ragnauth et al., 2010). When looking for Piezo1 expression specifically, lysates were run on a TruPAGE precast 4–20% gradient gel (Sigma-Aldrich) at 120 V for 2 h. Proteins were transferred onto PVDF membranes at 30 V for 3 h prior to the membrane being blocked in 5% milk in TBS containing 0.05% TWEEN 20. The following antibodies were used: anti-Piezo1 (1:500, Novus Biologicals, NBP1-78537, RRID:AB_11003149), anti-GAPDH (1:4000, Cell Signaling Technology, 2118, RRID:AB_561053), anti-acetylated α-tubulin (K40) (1:1000, Cell Signaling Technology, 3971, RRID:AB_2210204), anti-α-tubulin (total) (1:1000, Cell Signaling Technology, 3873, RRID:AB_1904178), anti-HDAC6 (D2E5) (1:1000, Cell Signaling Technology, 7558, RRID:AB_10891804), anti-rabbit HRP (1:2000, Sigma-Aldrich, GENA934, RRID:AB_2722659) and anti-mouse HRP (1:2000, Sigma-Aldrich, AP160P, RRID:AB_92531). Lysates from 12 and 72 kPa hydrogels were repeated in triplicate for each individual experimental repeat. Uncropped blots are shown in Fig. S7.

### Immunofluorescence and VSMC area and volume analysis

Cells were fixed in 4% paraformaldehyde for 10 min, permeabilised with 0.5% (v/v) NP40 for 5 min, then blocked with 3% (w/v) BSA in PBS for 1 h. Primary staining against lamin A/C (1:200, Sigma-Aldrich, SAB4200236, RRID:AB_10743057) was performed overnight at 4°C in 3% BSA in PBS. Secondary antibody staining was performed using the appropriate Alexa Fluor 488 antibody (1:400, Thermo Fisher Scientific, A-11001, RRID:AB_2534069) in the dark for 2 h. F-actin was visualised using Rhodamine Phalloidin (1:400, Thermo Fisher Scientific, R145). Images were captured at 20× magnification using a Zeiss LSM980 Airyscan confocal microscope. Cell area and volume was measured using FIJI open-source software as described previously (Johnson et al., 2024; Schindelin et al., 2012).

### Traction force microscopy

VSMCs were seeded onto polyacrylamide hydrogels containing 0.5 µm red fluorescent (580/605) FluoSpheres (1:1000, Invitrogen). Following angiotensin II stimulation (30 min), cell lysis was achieved by the addition of 0.5% (v/v) Triton X-100. Images were captured at 20× magnification before and after lysis at 2-min intervals using a Zeiss Axio Observer live-cell imaging system. Drift was corrected using the ImageJ StackReg plugin (https://imagej.net/plugins/stackreg) and traction force was calculated using an ImageJ plugin that measured FluoSphere displacement (Tseng et al., 2012). Briefly, bead displacement was measured using the first and last image of the movie sequence. The cell region was determined by overlaying the traction map with the phase image, selecting the cell traction region with an region of interest and extracting the traction forces in each pixel using the XY coordinate function in FIJI (Ahmed et al., 2022; Porter et al., 2020).

### Cold-stable microtubule stability assay

Cold-stable microtubules were identified as per previous studies (Atkinson et al., 2018). Following treatment, cells were placed on ice for 15 min before being washed once with PBS and twice with PEM buffer [80 μM PIPES pH 6.8, 1 mM EGTA, 1 mM MgCl_2_, 0.5% Triton X-100 and 25% (w/v) glycerol] for 3 min. Cells were fixed in ice-cold methanol for 20 min, then blocked with 3% BSA in PBS for 1 h. Microtubules were visualised by staining for α-tubulin (1:200, Cell Signalling Technology, 3873, RRID:AB_1904178), whereas cell nuclei were visualised using a mounting medium containing DAPI. Images were captured at 40× magnification using a Zeiss AxioPlan 2ie microscope. Quantification of the number of cold-stable microtubules was performed manually. Captured images were viewed at 300% magnification, a magnification level that enabled individual microtubules to be seen more clearly but without detrimental image pixelation. A microtubule was counted as a continuous strand of α-tubulin, whether it existed freely or part of as part of a network. If part of a network, a microtubule was deemed to end at the point where it contacted another microtubule. Strands shorter than 1 μm were excluded from our analysis, as were blob-like aggregations of tubulin.

### Cell viability assay

Cell viability was determined using a RealTime-Glo MT Cell Viability Assay (Promega), as per the manufacturer’s instructions. Briefly, 5000 cells per well were seeded in a 96-well plate and exposed to a range of drug concentrations for 1 h. Luminescence was subsequently measured using a Wallac EnVision 2103 Multilabel Reader (PerkinElmer).

### Fluo-4 Ca^2+^ imaging

Cells were seeded on 12 and 72 kPa hydrogels and incubated in basal medium for 48 h. Cells were loaded with 3 µM Fluo-4 AM (Thermo Fisher Scientific, F14201) diluted in basal medium for 30 min. Paclitaxel, colchicine and tubastatin were used at working concentrations listed in Table S1. Cells were co-treated with vehicle control or compounds of interest during the Fluo-4-loading step. Cells were washed in PBS and returned to basal medium prior to imaging. Cells were placed onto a Zeiss Axiovert 200M inverted microscope stage and images were captured every 500 ms. Cells were imaged for 5 min prior to angiotensin II (10 µM) stimulation. Cells were subsequently imaged for a further 20 min after addition of angiotensin II. Finally, the ionophore A23187 (20 µM, Tocris) was added for a further 5 min to confirm that the Ca^2+^ imaging had worked. Images were analysed in ImageJ by selecting a region of interest and extracting the fluorescence intensities (F) for each time point using the ‘plot Z-axis profile’ option. The background was subtracted and the ΔF and ΔF/F_0_ values were calculated and analysed using GraphPad prism.

### Statistical analysis

Statistical analysis was performed using GraphPad Prism 9. Results are presented as mean±s.e.m., with individual data points shown. The number of independent repeats performed and total number of cells analysed per experiment are detailed in the corresponding figure legends. All statistical analyses throughout the manuscript were performed on the means of each independent experimental repeat. To compare more than two conditions, a one-way ANOVA was performed with a Tukey's multiple comparison post hoc test. Concentration response curves are presented as mean±s.e.m. plotted on a semi-logarithmic scale. Comparisons between concentration ranges on different hydrogel stiffness were performed using a two-way ANOVA followed by Sidak's post hoc test. Differences between conditions were considered statistically significant when *P*<0.05.

## Supplementary Material



10.1242/joces.262310_sup1 Supplementary information
