# Supplementary material for: A microtubule stability switch alters isolated vascular smooth muscle Ca2+ flux in response to matrix rigidity
Source: J Cell Sci. 2024 Nov 12;137(21):jcs262310. doi: 10.1242/jcs.262310 (PMC11586521; doi:10.1242/jcs.262310)
Supplement: Supplementary information [file joces-137-262310-s1.pdf]

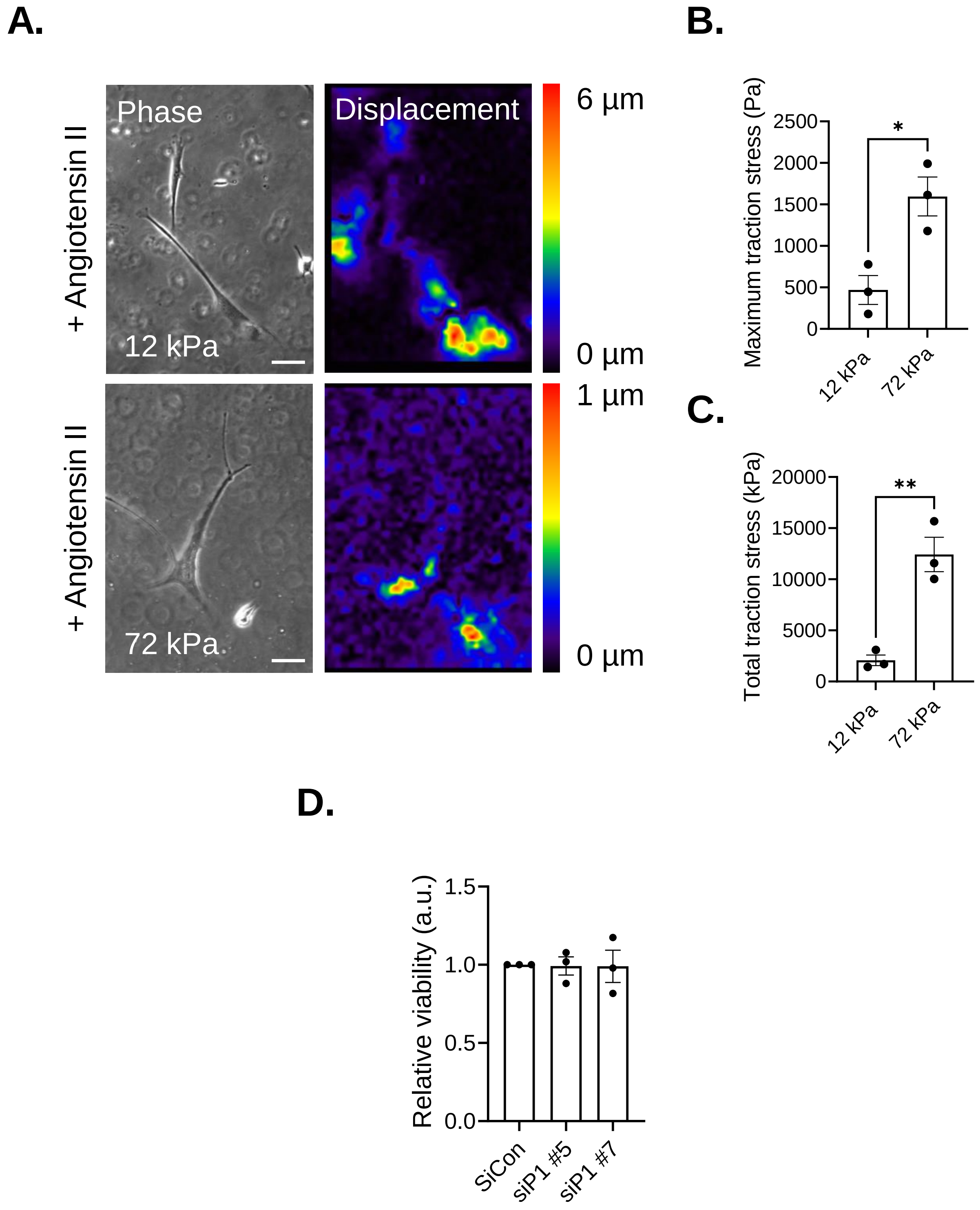

Fig. S1. VSMCs generate enhanced traction stress on rigid hydrogels. A) Representative phase images and bead displacement heat maps of AngII (10 µM) stimulated VSMCs cultured on 12 or 72 kPa polyacrylamide hydrogels. Scale bar = 100 µm. Graphs show **B)** maximum and **C)** total traction stress generation, representative of 3 independent experiments, with ≥38 cells analysed per condition. Black dots represent mean data for each individual experimental repeat. Significance determined using unpaired student t-Test. * = $p < 0.05$ and ** = $p < 0.01$. Error bars represent ± SEM. **D)** Graph shows relative VSMC viability following a 72-hour treatment with scrambled (siCon) or piezo1-targeting (siP1 #5/#7) siRNA. Black dots represent mean values for each independent experimental repeats performed in triplicate. One-way ANOVA followed by Tukey's test determined that no significant differences existed between the experimental groups. Error bars represent ± SEM).

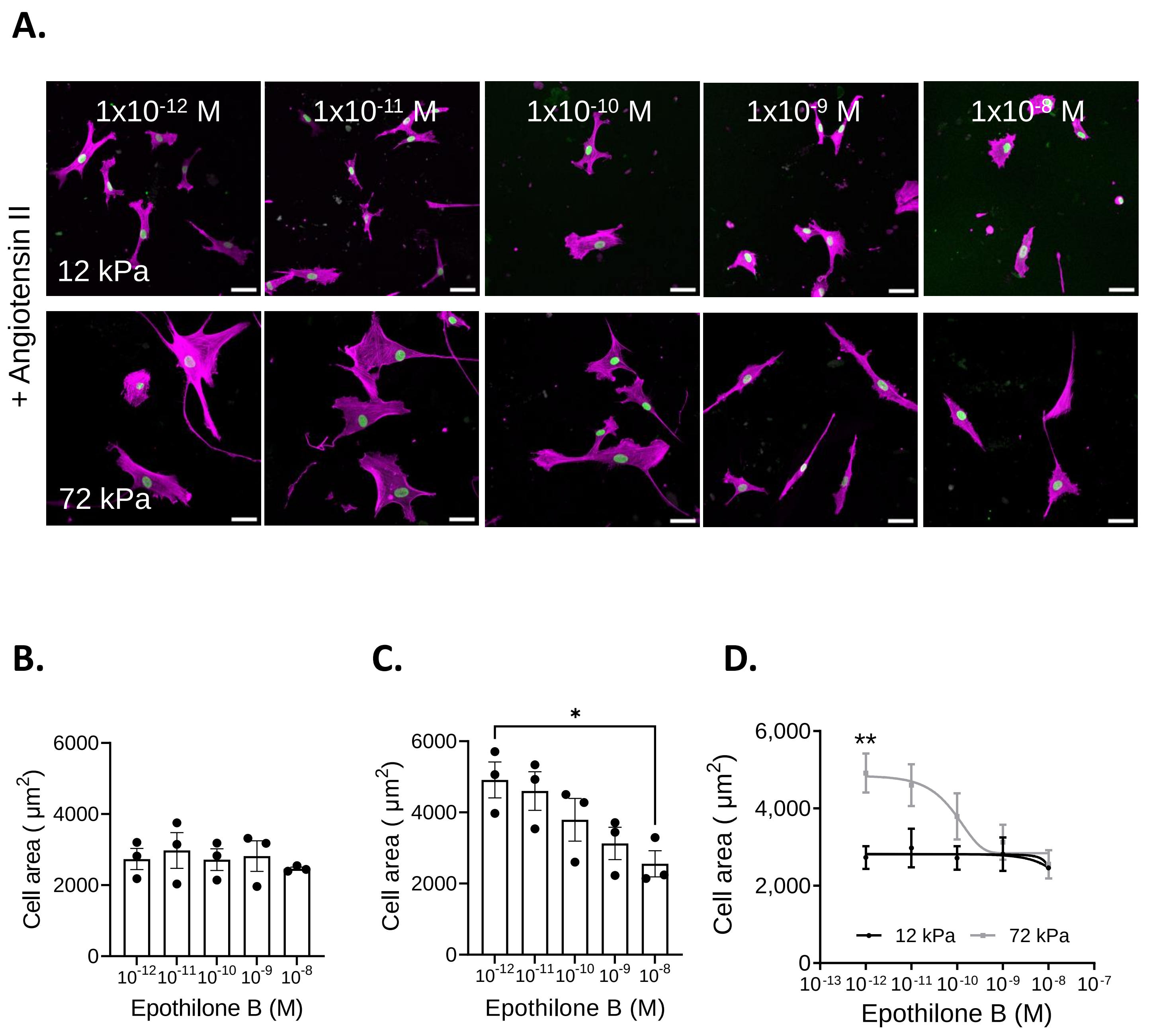

Fig. S2. Epothilone treatment blocks increased VSMC area response on rigid hydrogels.
A) Representative images of VSMCs pre-treated with increasing concentrations of epothilone B, prior to angiotensin II stimulation, on 12 kPa and 72 kPa hydrogels. Purple = Rhodamine Phalloidin, green = DAPI and Scale bar = 50 µm. Graphs show VSMC area on **B)** 12 kPa and **C)** 72 kPa hydrogels representative of 3 independent experiments with ≥53 cells analysed per condition. Black dots represent mean data from each individual experimental repeat. Significance determined using a one-way ANOVA followed by Tukey's test.
D) Comparison of VSMC response to epothilone B pre-treatment on 12 and 72 kPa hydrogels. Data is expressed as the mean of the means calculated from 3 independent experiments; significance determined using a two-way ANOVA followed by Sidak's test. (* = $p < 0.05$ and ** = $p < 0.01$. Error bars represent ± SEM).

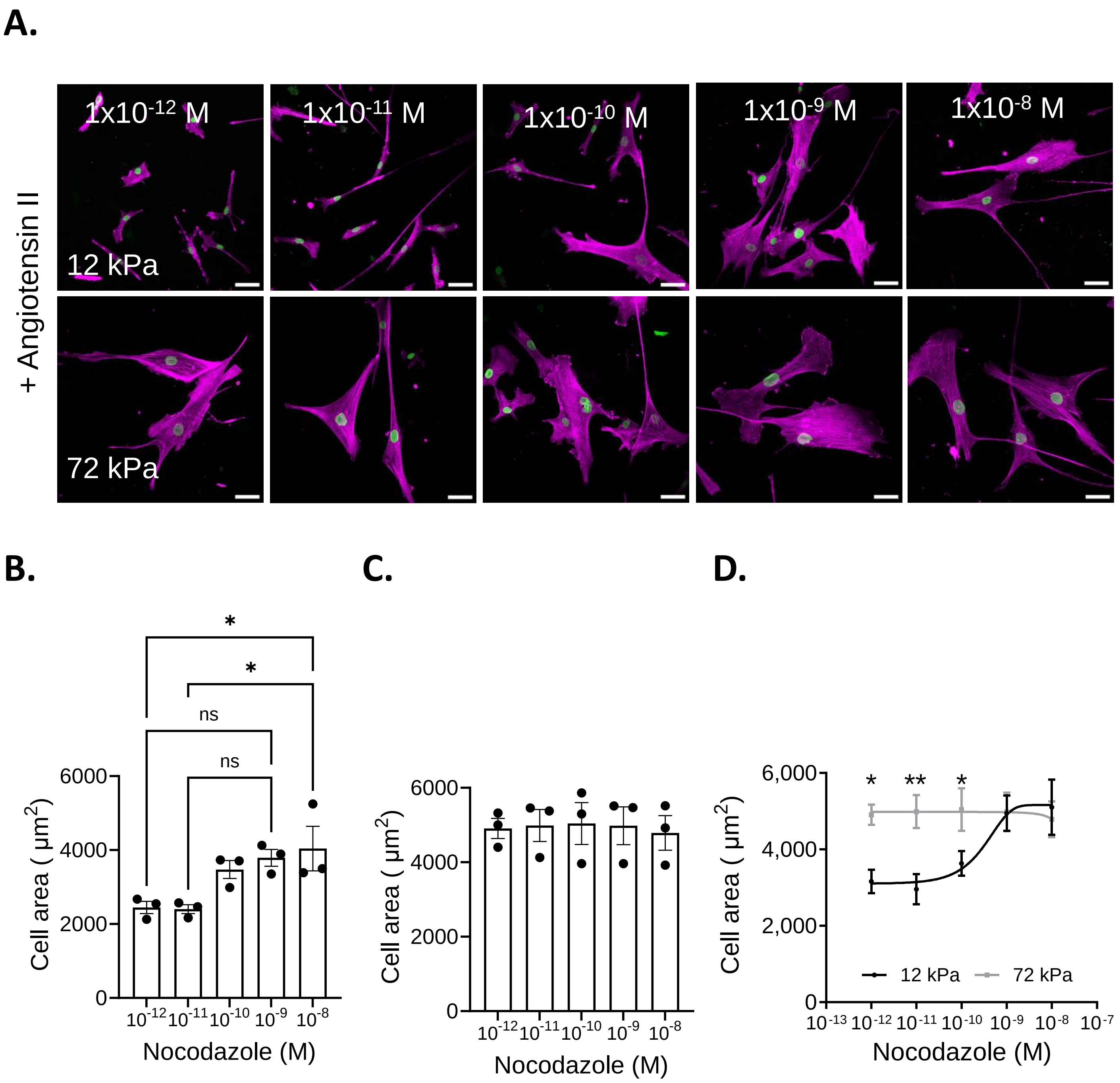

Fig. S3. Nocodazole treatment increases VSMC area on pliable hydrogels. A) Representative images of VSMCs pre-treated with increasing concentrations of nocodazole, prior to angiotensin II stimulation, on 12 kPa and 72 kPa hydrogels. Purple = Rhodamine Phalloidin, green = DAPI and Scale bar = 50 µm. Graphs show VSMC area on **B)** 12 kPa and **C)** 72 kPa hydrogels representative of 3 independent experiments with ≥58 cells analysed per condition. Black dots represent mean data from each individual experimental repeat. Significance determined using a one-way ANOVA followed by Tukey's test. **D)** Comparison of VSMC response to nocodazole pre-treatment on 12 and 72 kPa hydrogels. Data is expressed as the mean of the means calculated from 3 independent experiments; significance determined using a two-way ANOVA followed by Sidak's test. (* = p < 0.05 and ** = p <0.01. Error bars represent ± SEM).

Journal of Cell Science • Supplementary information

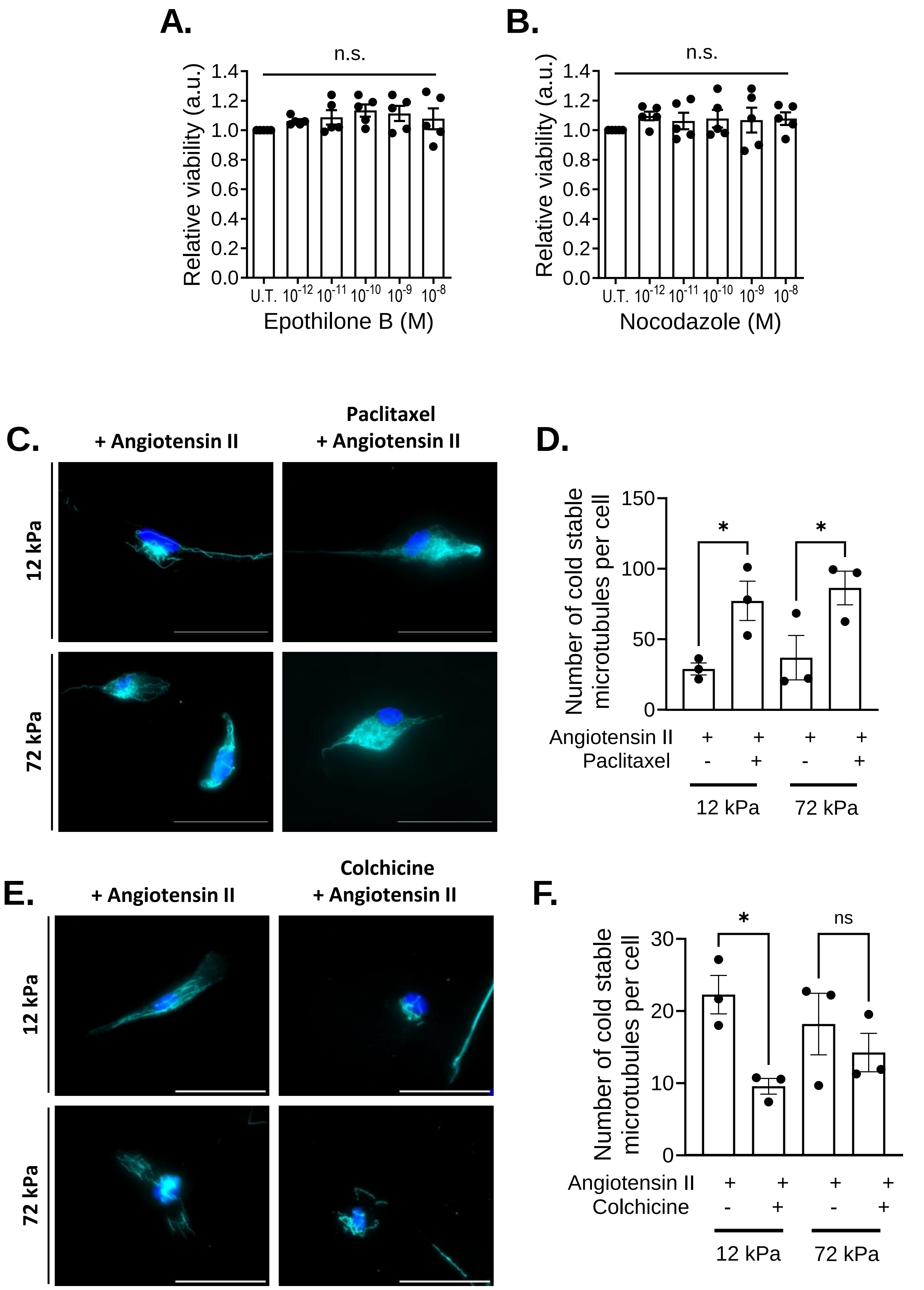

Fig. S4. Microtubule targeting agents regulate microtubule stability in isolated VSMCs. Relative VSMC viability following a 1-hour treatment with increasing concentrations of **A)** epothilone B and **B)** nocodazole. U.T. = Untreated. Data is representative of 5 independent experiments; significance determined using a one-way ANOVA followed by Tukey's test. (n.s. = *non-significant*, error bars represent ± SEM). Representative images of isolated VSMCs cultured on 12 or 72 kPa polyacrylamide hydrogels pretreated with **C)** paclitaxel or **E)** colchicine prior to angiotensin II stimulation. Cold-stable microtubules, (α-tubulin, aqua) and nuclei (DAPI, blue). Scale bar = 50 μm. Graphs show number of cold-stable microtubules per cell after **D)** paclitaxel treatment, representative of 3 independent experiments, with ≥65 cells analysed per condition; and **F)** colchicine treatment, representative of 3 independent experiments, with ≥82 cells analysed per condition. Black dots represent mean data for each individual experimental repeat. Significance determined using a one-way ANOVA followed by Sidak's test. (* = $p < 0.05$, error bars represent ± SEM).

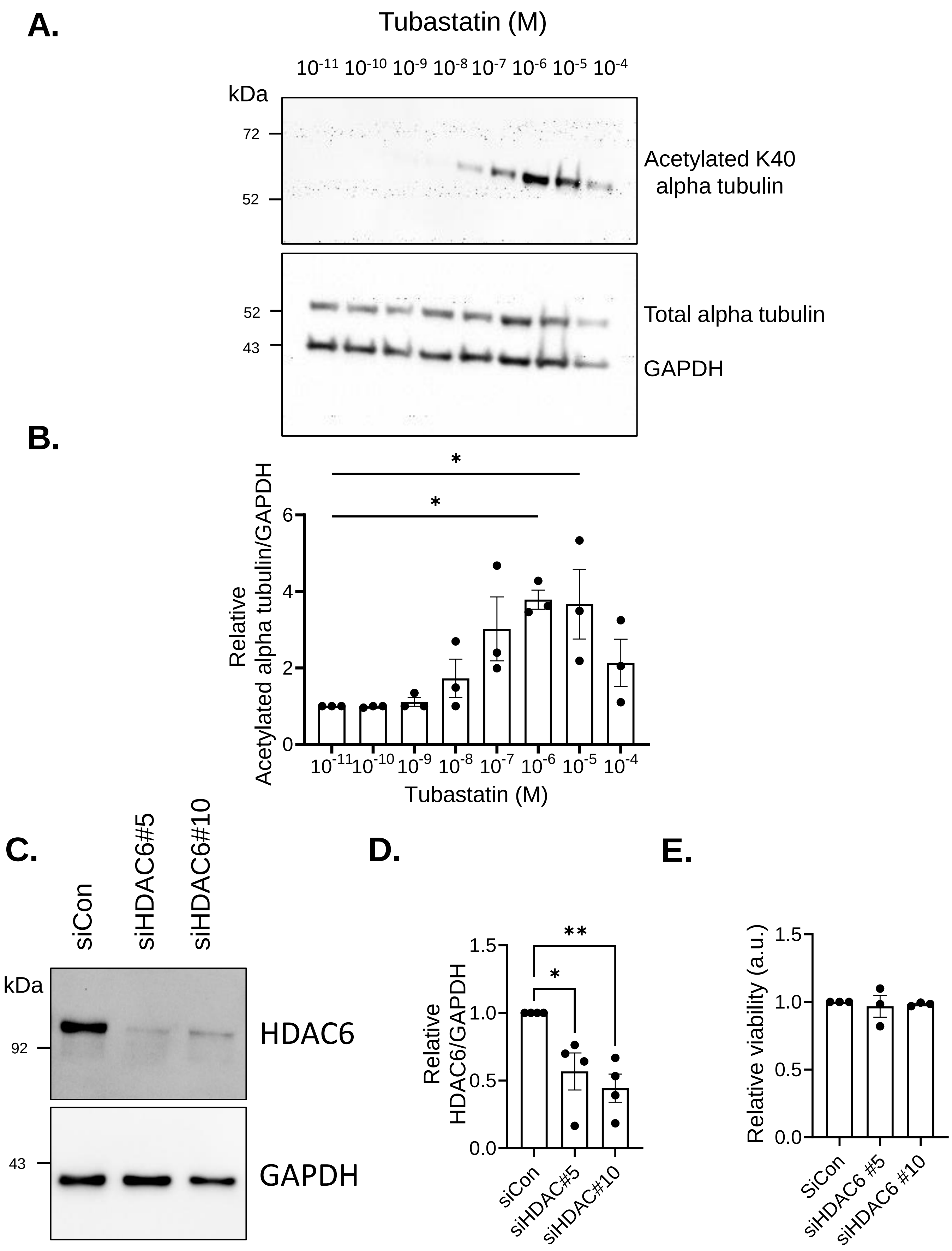

Fig. S5. Conformation of tubastatin function and HDAC6 depletion in VSMCs. A) Representative WB of acetyl-alpha tubulin levels in isolated VSMCs treated with a concentration range of tubastatin for 1 hour. **B)** Graph shows the relative acetyl-alpha tubulin/GAPDH level determine by densitometry and represents the combined data of 3-independent experiments. Black dots represent the mean data of each individual experimental repeat. Significance determined using a one-way ANOVA followed by Sidak's test. (* = $p < 0.05$, error bars represent ± SEM). **C)** Representative WB of HDAC6 levels in scrambled (siCon) and siHDAC6 specific (siHDAC6#5 and siHDAC6#10) siRNA transfected VSMCs. **D)** Graph shows the relative HDAC6/GAPDH level determined by densitometry and represents the combined data of 4-independent experiments. Black dots represent the mean data of each individual experimental repeat. Significance determined using a one-way ANOVA followed by Sidak's test. (* = $p < 0.05$, ** = $p < 0.01$ error bars represent ± SEM). Relative VSMC viability following a 72-hour treatment with scrambled (siCon) or HDAC6-targeting (siHDAC6 #5/#10) siRNA. Black dots represent mean values for each independent experimental repeats performed in triplicate. **E)** Graph shows relative VSMC viability following a 72-hour treatment with scrambled (siCon) or HDAC6-targeting (siHDAC6 #5/#10) siRNA. Black dots represent mean values for each independent experimental repeats performed in triplicate. One-way ANOVA followed by Tukey's test determined that no significant differences existed between the experimental groups. Error bars represent ± SEM).

Journal of Cell Science • Supplementary information

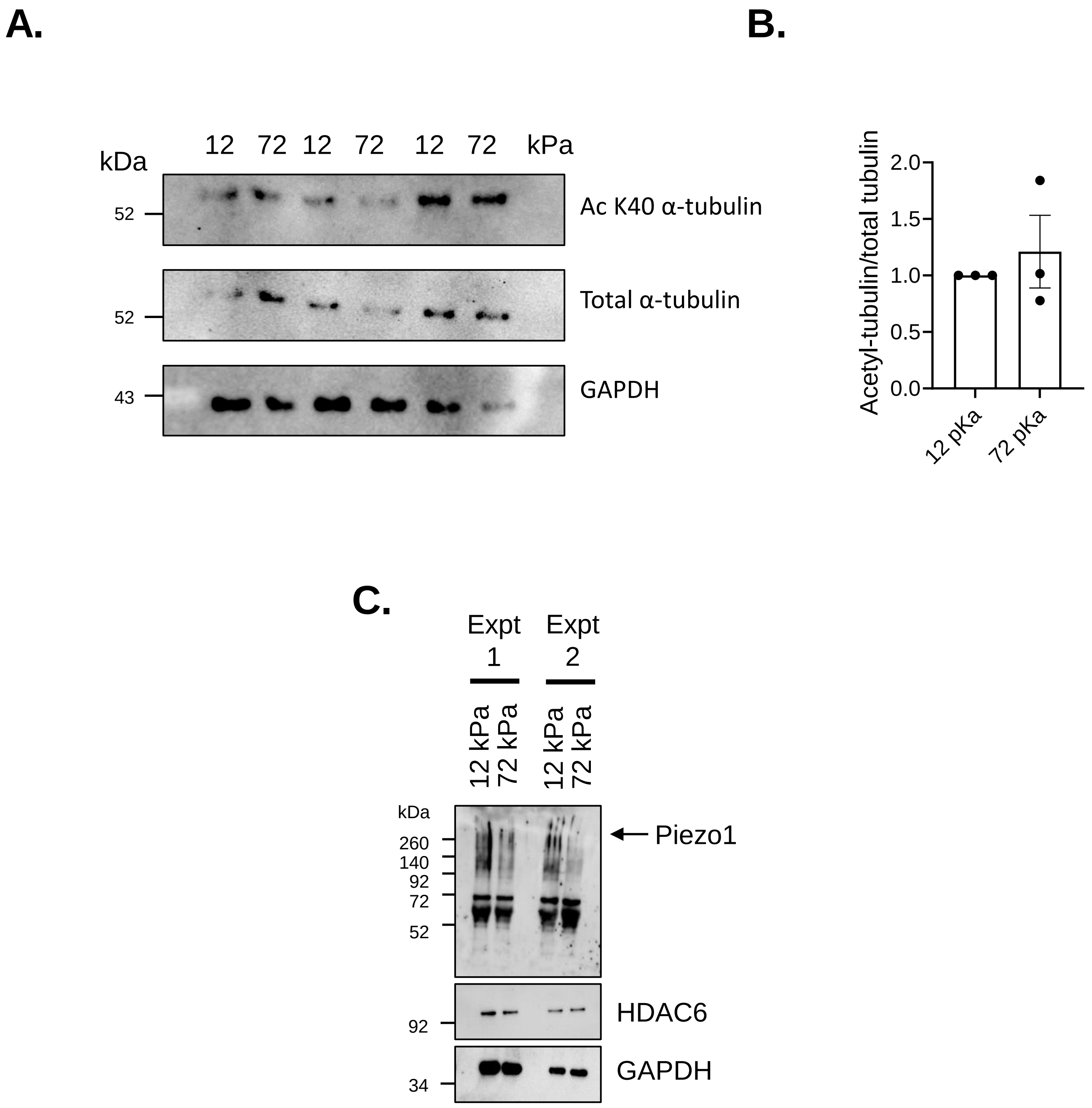

Fig. S6. Matrix stiffness does not alter acetyl-alpha tubulin levels in VSMCs. A) Graph shows the relative acetyl-alpha tubulin/total tubulin level determine by densitometry and represents the combined data of 3-independent experiments. Black dots represent the mean data of each individual experimental repeat. Significance determined using a one-way ANOVA followed by Sidak's test. (* = $p < 0.05$, error bars represent ± SEM). **B)** WB of piezo1 and HDAC6 levels in VSMCs grown on 12 and 72 kPa hydrogels for 3 days. Each experiment (expt 1 and 2) represents an independent experiment. **C)** Representative WB of acetyl-alpha tubulin levels in isolated VSMCs grown on 12 and 72 kPa hydrogels for 3 days.

Journal of Cell Science • Supplementary information

Fig. 1C uncropped WBs. Some lanes removed as loaded with lysates that are not part of this study. This extended image contains an untransfected control and an addition piezo1 targeting siRNA (siP#10)

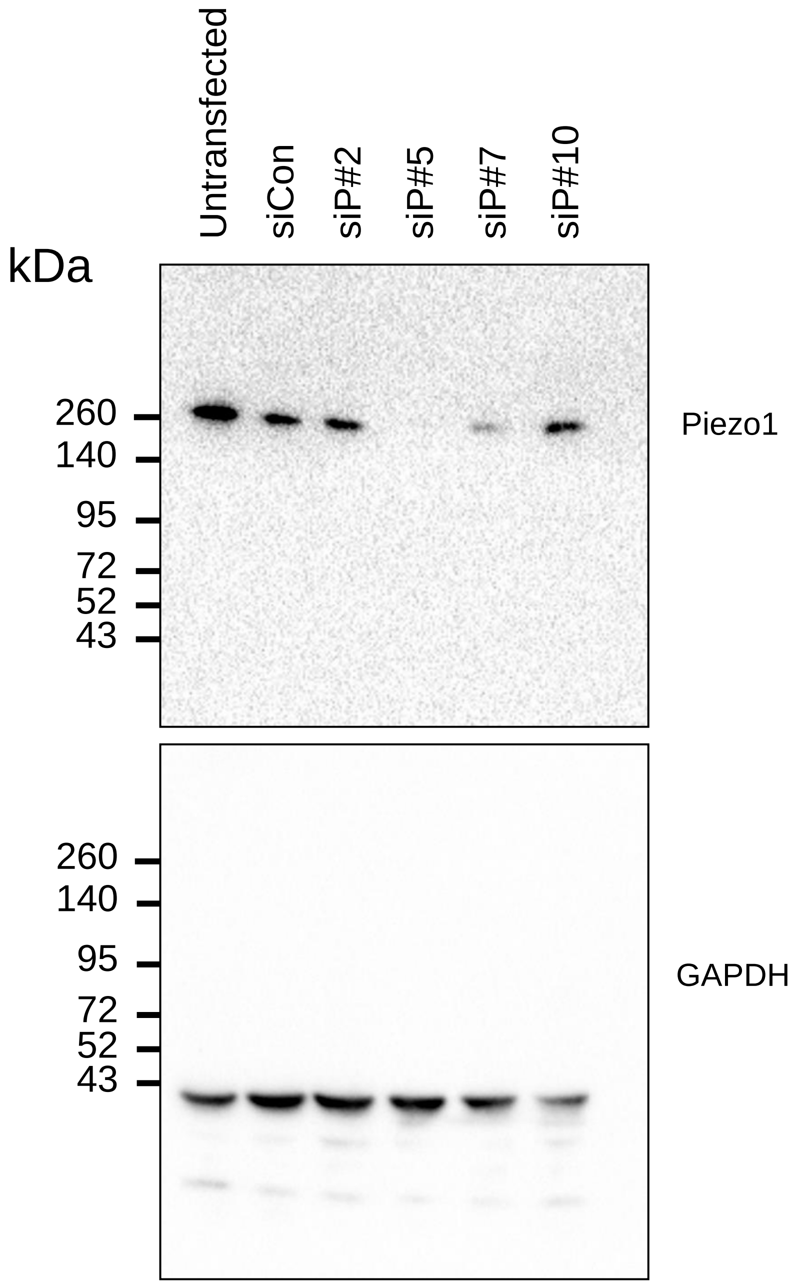

Journal of Cell Science • Supplementary information

Fig. S5A uncropped WBs.

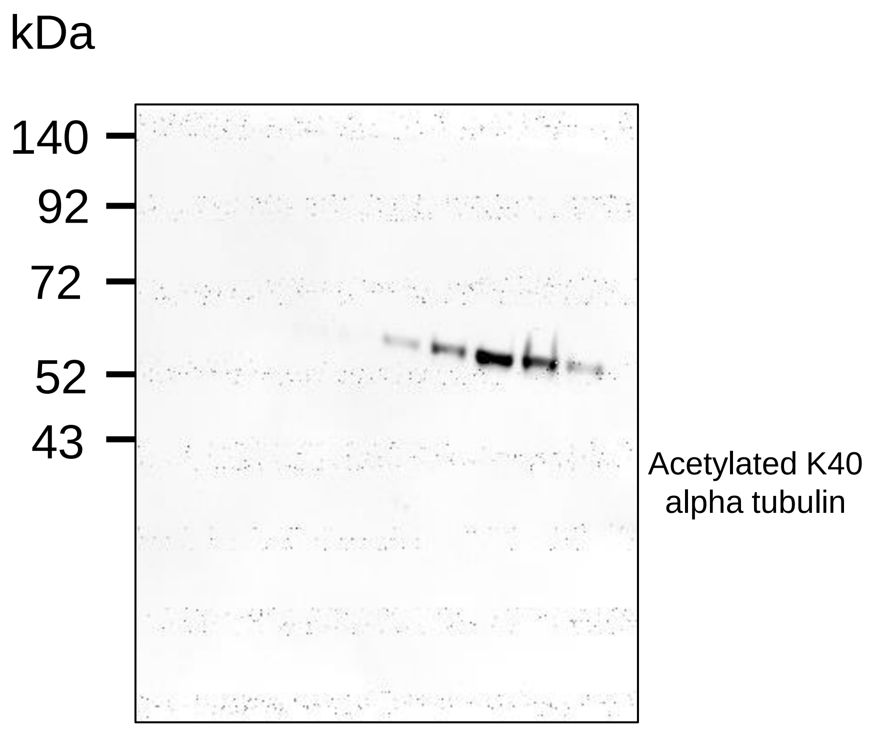

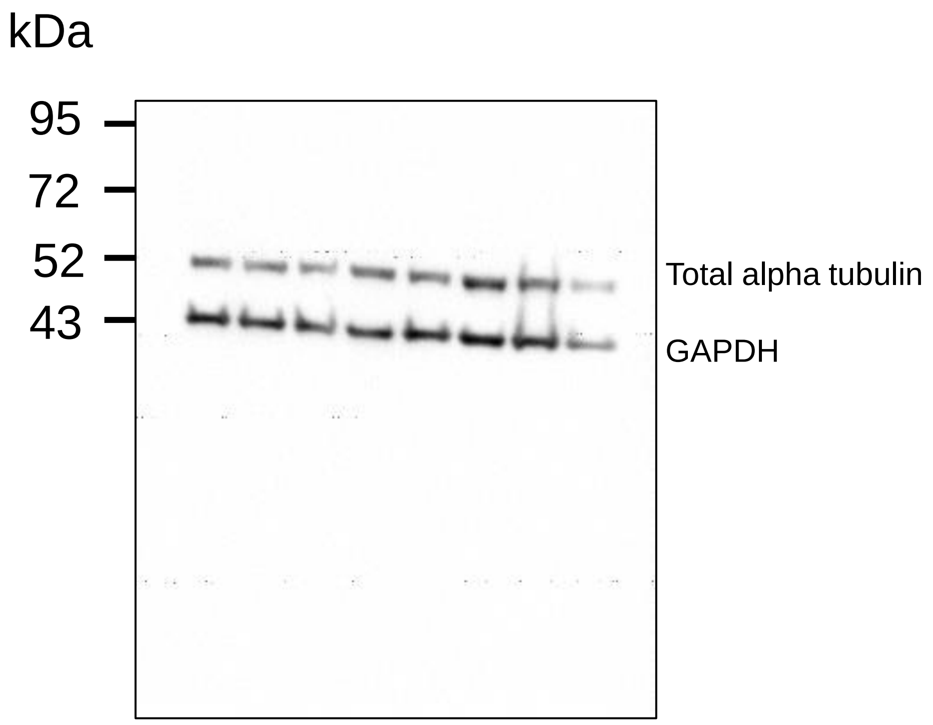

Journal of Cell Science • Supplementary information

Fig. S5C uncropped WB. Some lanes removed as loaded with lysates not part of this study.

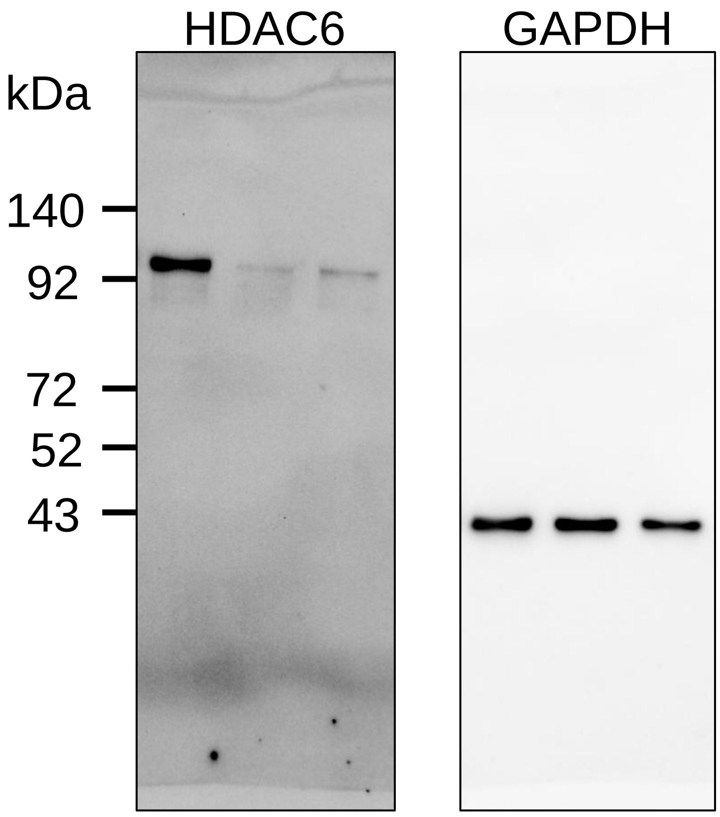

Journal of Cell Science • Supplementary information

Fig. S6A uncropped WBs.

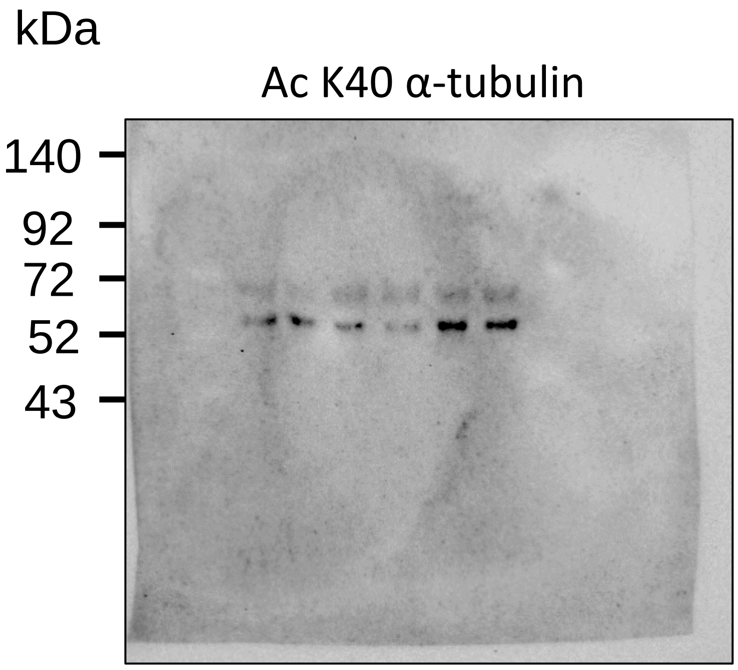

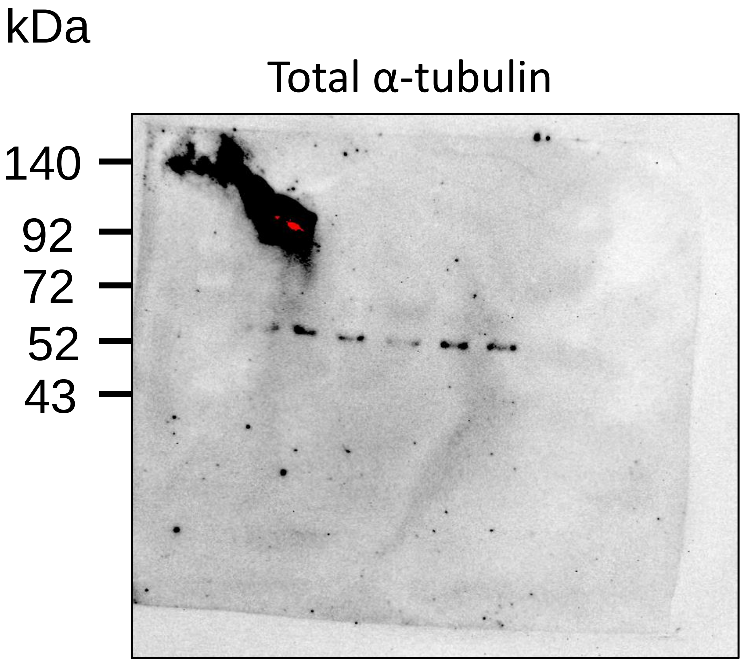

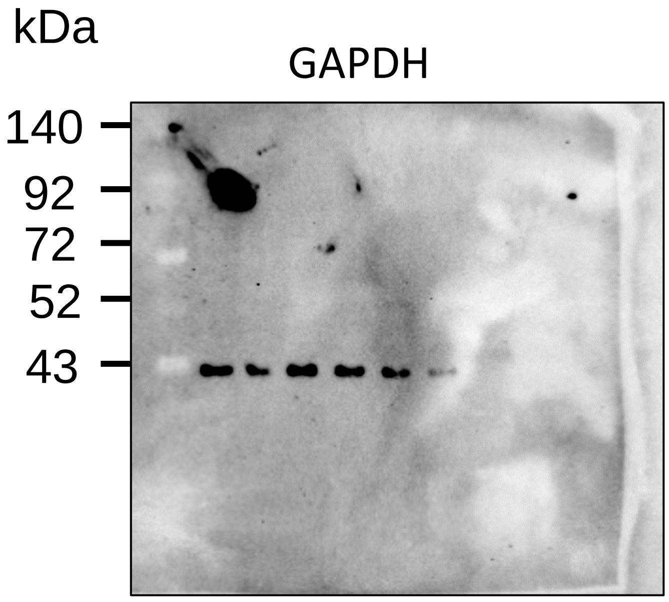

Fig. S6C uncropped WBs.

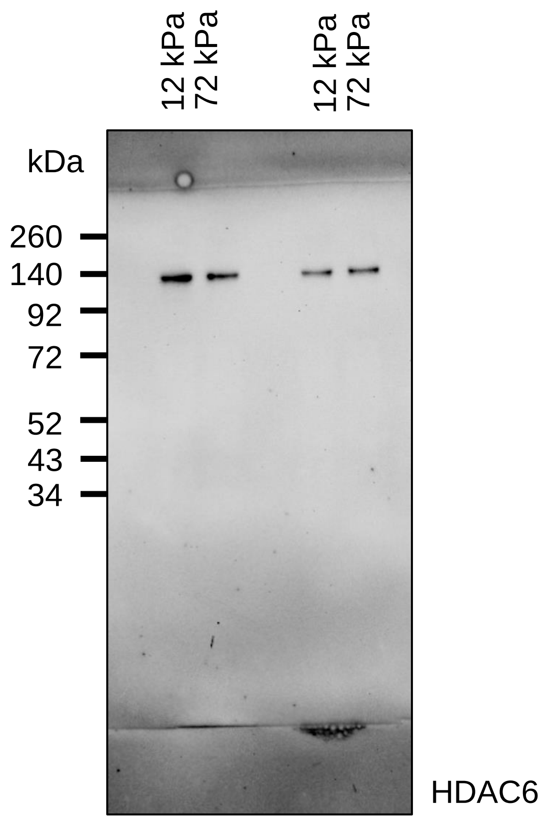

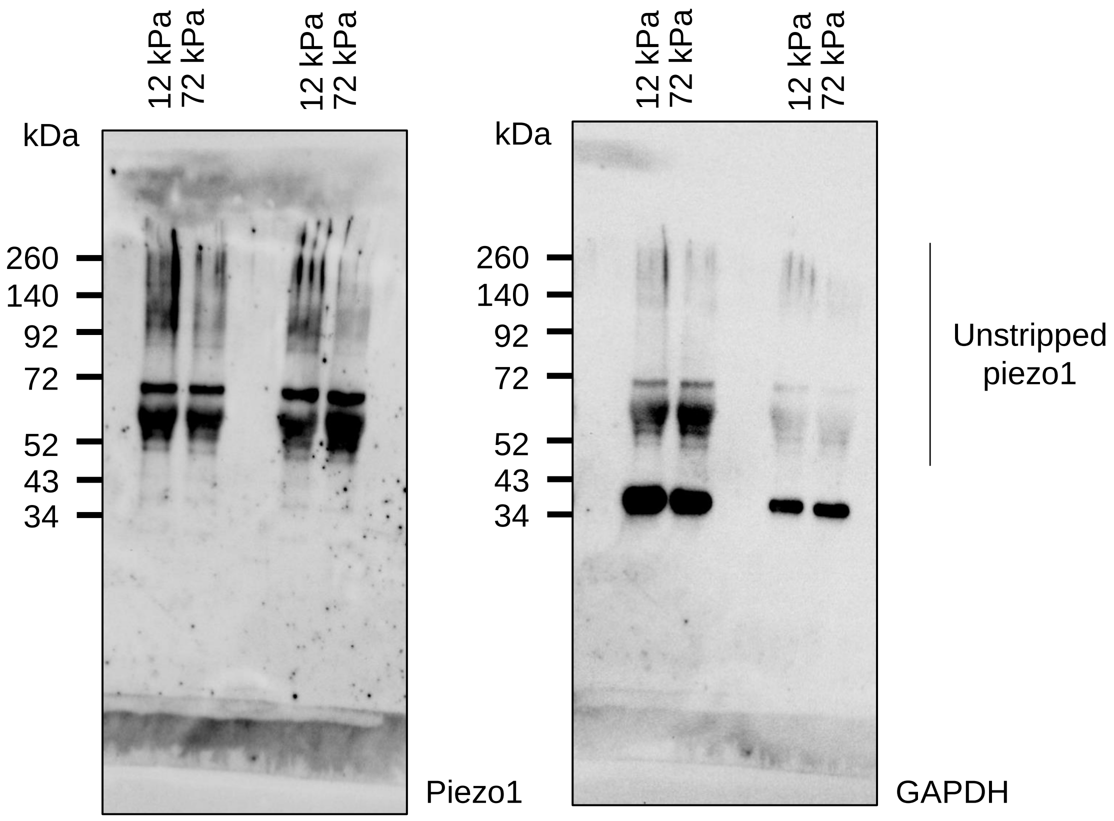

Fig. S7. Blot transparency

Table S1. Concentrations and details of compounds used in this study

Compound	Product Code	Supplier	Vehicle	Concentration Range Tested	Working Concentration
Angiotensin II	A9525	Merck	dH_2O		10 µM
Colchicine	C9754	Merck	dH_2O	0.1 - 1000nM	100 nM
Paclitaxel	T7402	Merck	dH_2O	0.001 - 10 nM	1 nM
Nocodazole	ab120630	Abcam	DMSO	0.001 - 10 nM	-
Epothilone B	E2656	Merck	DMSO	0.001 - 10 µM	-
Tubastatin A	#6270	Tocris	DMSO	0.01 - 1 µM	1 µM

Journal of Cell Science • Supplementary information
