# Supplementary material for: A microtubule stability switch alters isolated vascular smooth muscle Ca2+ flux in response to matrix rigidity
Source: J Cell Sci. 2024 Nov 12;137(21):jcs262310. doi: 10.1242/jcs.262310 (PMC11586521; doi:10.1242/jcs.262310)
Supplement: Supplementary file 1 [file joces-137-262310_review_history.pdf]

Journal of Cell Science

A microtubule stability switch alters isolated vascular smooth muscle Ca^{2+} flux in response to matrix rigidity

Robert T. Johnson, Finn Wostear, Reesha Solanki, Oliver Steward, Alice Bradford, Christopher Morris, Stefan Bidula and Derek T. Warren
DOI: 10.1242/jcs.262310

Editor: Andrew Ewald

Review timeline
Original submission: 29 May 2024
Editorial decision: 2 July 2024
First revision received: 10 September 2024
Accepted: 15 September 2024

Original submission

First decision letter

MS ID#: JOCES/2024/262310

MS TITLE: A microtubule stability switch alters isolated vascular smooth muscle calcium flux in response to matrix rigidity.

AUTHORS: Robert T Johnson, Finn Wostear, Reesha Solanki, Oliver Steward, Christopher Morris, Stefan Bidula, and Derek T Warren
ARTICLE TYPE: Research Article

We have now reached a decision on the above manuscript.

To see the reviewers' reports and a copy of this decision letter, please go to: https://submit-jcs.biologists.org and click on the 'Manuscripts with Decisions' queue in the Author Area. (Corresponding author only has access to reviews.)

As you will see, the reviewers gave favourable reports but raised some critical points that will require amendments to your manuscript. I hope that you will be able to carry these out because I would like to be able to accept your paper, depending on further comments from reviewers.

Please ensure that you clearly highlight all changes made in the revised manuscript. Please avoid using 'Tracked changes' in Word files as these are lost in PDF conversion.

I should be grateful if you would also provide a point-by-point response detailing how you have dealt with the points raised by the reviewers in the 'Response to Reviewers' box. Please attend to all of the reviewers' comments. If you do not agree with any of their criticisms or suggestions please explain clearly why this is so.

Reviewer 1

Advance summary and potential significance to field

This is a well written paper that provides evidence for an important role of microtubule stability in directing VSMC responses to matrix stiffness. The authors have built on their previous work showing

© 2024. Published by The Company of Biologists under the terms of the Creative Commons Attribution License (https://creativecommons.org/licenses/by/4.0/).

that increased ECM stiffness promotes VSMC dysfunction by increasing volume. Here they demonstrate that this is due to Piezo-mediated calcium influx which destabilises microtubules. The data is clearly presented and described throughout and makes an important contribution to our understanding of VSMC mechanobiology and VSMC dysfunction in ageing and cardiovascular disease. Importantly, the paper also highlights the potential for manipulation of microtubule dynamics as a possible therapeutic target. A lot of mechanistic data is provided which strengthens the manuscript.

I have only very minor comments:

Comments for the author

1. The data on cold-stable microtubules are interesting. Please can you add details to the relevant methods section explaining how these images were quantified. It would strengthen the manuscript to include images taken at higher magnification if possible.

2. Please add a label to Figure 5F-H outlining the gel stiffness

Reviewer 2

Advance summary and potential significance to field

The manuscript describes some interesting and novel findings on the role of ECM compliance in the regulation of VSMC volume, via Piezo1-mediated calcium signaling and changes in microtubule stability. The manuscript is very well written and the work has been conducted to a high level. The data presented extends our understanding of the novel mechanisms underlying the biomechanical regulation of VSMC function and behaviour in healthy and diseased blood vessels.

Comments for the author

I only have a few relatively minor comments:

1 One of the main endpoints measured is the volume of VSMC cultured on substrates of varying compliance. Increased volume is an important feature of hypertrophic cells, as the authors state in the introduction. However, it is not clear if the changes in volume observed are indicative of VSMC hypertrophy. It may be helpful if the authors could add a short discussion of the pathophysiological significance of this volume change. Are other hypertrophic features (increased protein synthesis?) also observed in cells on stiffer substrates?

2 Can the authors clarify how some of the statistical analysis was performed? In some figures (e.g. Figure 1E siCon vs siP#5) the data is marked as significantly different, although the overlapping error bars and small difference in means doesn't look like it would be significant. Was the statistical analysis performed on the three mean values obtained from each experiment or by treating every individual cell as a separate n?

3 It may be helpful if the authors can provide a more detailed description of how the image analysis was performed for the cold-stable microtubule assay.
In some of the images the cells are quite small and it is hard to imagine how one would count individual tubules.

4 The authors state in the discussion that they show that increased microtubule K40 acetylation stimulated both enhanced calcium flux and VSMC volume. This is an over-interpretation of the data. They show that HDAC6 is required for K40 acetylation and is associated with the two metrics but have not presented data that demonstrates a direct functional link between tubulin K40 acetylation and Calcium flux and volume. Can the authors modify this statement?

Reviewer 3

© 2024. Published by The Company of Biologists under the terms of the Creative Commons Attribution License (https://creativecommons.org/licenses/by/4.0/).

Advance summary and potential significance to field

This manuscript reports on the mechanism of VSMC response to stiffness. It finds that microtubule stability in VSMCs is reduced in response to enhanced matrix rigidity via piezo1-mediated Ca2+ influx. This involves the microtubule deacetylase HDAC6 which regulated K40 alpha tubulin acetylation, VSMC volume and Ca2+ flux in response to stiffness. Overall, data are high quality and the manuscript is well written

Specific comments

Roles for Piezo1 and HDAC6 are identified – I would like to know if plating on different stiffnesses alters the expression of PIEZO1 and HDAC6 either by qPCR or ideally at the protein level

Do the Piezo1 KD, HDAC KD alter cell viability? Live/dead stain would be useful

Comments for the author

Roles for Piezo1 and HDAC6 are identified – I would like to know if plating on different stiffnesses alters the expression of PIEZO1 and HDAC6 either by qPCR or ideally at the protein level

Do the Piezo1 KD, HDAC KD alter cell viability? Live/dead stain would be useful

---

First revision

Author response to reviewers' comments

We thank the Reviewers for their supportive and positive comments on our manuscript. We have addressed all the minor comments raised by the Reviewers and we believe that these tweaks have increased the clarity and robustness of our findings.

Reviewer 1 Advance Summary and Potential Significance to Field:
This is a well written paper that provides evidence for an important role of microtubule stability in directing VSMC responses to matrix stiffness. The authors have built on their previous work showing that increased ECM stiffness promotes VSMC dysfunction by increasing volume. Here they demonstrate that this is due to Piezo-mediated calcium influx which destabilises microtubules. The data is clearly presented and described throughout and makes an important contribution to our understanding of VSMC mechanobiology and VSMC dysfunction in ageing and cardiovascular disease. Importantly, the paper also highlights the potential for manipulation of microtubule dynamics as a possible therapeutic target. A lot of mechanistic data is provided which strengthens the manuscript. I have only very minor comments:

Reviewer 1 Comments for the Author:
1.The data on cold-stable microtubules are interesting. Please can you add details to the relevant methods section explaining how these images were quantified. It would strengthen the manuscript to include images taken at higher magnification if possible.

Thanks for these important suggestions.

We have now changed the colours on the images throughout. Microtubules are now in grey, making them easier to see against the black background. Representative images now also have increased size, we have zoomed in on the cells. We believe that these changes have increased the clarity of these figures.

More detail describing the analysis steps has now been added to the methods section, which now reads:

© 2024. Published by The Company of Biologists under the terms of the Creative Commons Attribution License (https://creativecommons.org/licenses/by/4.0/).

lines 528 – 535

'Quantification of the number of cold-stable microtubules was performed manually. Captured images were viewed at 300% magnification, a magnification level that enabled individual microtubules to be seen more clearly but without detrimental image pixelation. A microtubule was counted as a continuous strand of a-tubulin, whether it existed freely or part of as part of a network. If part of a network, a microtubule was deemed to end at the point where it contacted another microtubule. Stands shorter than 1 μm were excluded from our analysis, as were blob-like aggregations of tubulin.'

2.Please add a label to Figure 5F-H outlining the gel stiffness

Sorry for this omission. This has now been added to Figures 5F-H.

*****

Reviewer 2 Advance Summary and Potential Significance to Field:
The manuscript describes some interesting and novel findings on the role of ECM compliance in the regulation of VSMC volume, via Piezo1-mediated calcium signaling and changes in microtubule stability. The manuscript is very well written and the work has been conducted to a high level. The data presented extends our understanding of the novel mechanisms underlying the biomechanical regulation of VSMC function and behaviour in healthy and diseased blood vessels.

Reviewer 2 Comments for the Author:

I only have a few relatively minor comments:

1 One of the main endpoints measured is the volume of VSMC cultured on substrates of varying compliance. Increased volume is an important feature of hypertrophic cells, as the authors state in the introduction. However, it is not clear if the changes in volume observed are indicative of VSMC hypertrophy. It may be helpful if the authors could add a short discussion of the pathophysiological significance of this volume change. Are other hypertrophic features (increased protein synthesis?) also observed in cells on stiffer substrates?

These are very important considerations that we do not currently know the answer to. To highlight these, we have added a discussion of the pathophysiological significance and current unknowns into the Discussion section, which now reads:

lines 410-418

'VSMC hypertrophy is an early indicator of dysfunction. Hypertrophy comprises both an increase in cell volume and increase in protein synthesis. It remains unknown whether the increased volume response of VSMCs on rigid hydrogels is accompanied by increased protein synthesis. It also remains unknown whether increase VSMC volume contributes to decrease aortic compliance further. Given that water is essentially incompressible under normal conditions, increased VSMC volume is likely to contribute to the pathophysiological aortic wall biomechanics. More research is needed to address these current gaps in our knowledge.'

2 Can the authors clarify how some of the statistical analysis was performed? In some figures (e.g. Figure 1E siCon vs siP#5) the data is marked as significantly different, although the overlapping error bars and small difference in means doesn't look like it would be significant. Was the statistical analysis performed on the three mean values obtained from each experiment or by treating every individual cell as a separate n?

© 2024. Published by The Company of Biologists under the terms of the Creative Commons Attribution License (https://creativecommons.org/licenses/by/4.0/).

We have double checked all statistical analyses performed in this manuscript. Despite the overlapping SEM error bars in Figure 1E, the data still passes the 0.05 cut off to be significant by two-way ANOVA, as described in the figure legend. All analyses are performed on the mean values obtained from 3-independent repeat experiments (minimum). We have now improved the clarity of our statistics section in the Methods. Now reads:

Lines 564-566

‘All statistical analyses throughout the manuscript were performed on the means of each independent experimental repeat.’

3 It may be helpful if the authors can provide a more detailed description of how the image analysis was performed for the cold-stable microtubule assay. In some of the images the cells are quite small and it is hard to imagine how one would count individual tubules.

We thank the reviewer for highlighting this limitation. We have now expanded the methods section to include a detailed description of how the analysis was performed. Please see Reviewer 1 response for complete description.

4 The authors state in the discussion that they show that increased microtubule K40 acetylation stimulated both enhanced calcium flux and VSMC volume. This is an over-interpretation of the data. They show that HDAC6 is required for K40 acetylation and is associated with the two metrics but have not presented data that demonstrates a direct functional link between tubulin K40 acetylation and Calcium flux and volume. Can the authors modify this statement?

Thank you for highlighting this, we completely agree that this is an overstatement. We have now removed this statement and altered the discussion to read:

Lines 382-384

‘Our findings suggests that changes in microtubule post-translational modification can influence Ca^{2+} flux and VSMC volume on pliable hydrogels.’

*****

Reviewer 3 Advance Summary and Potential Significance to Field:
This manuscript reports on the mechanism of VSMC response to stiffness. It finds that microtubule stability in VSMCs is reduced in response to enhanced matrix rigidity via piezo1-mediated Ca2+ influx. This involves the microtubule deacetylase HDAC6 which regulated K40 alpha tubulin acetylation, VSMC volume and Ca2+ flux in response to stiffness. Overall, data are high quality and the manuscript is well written

Specific comments

Roles for Piezo1 and HDAC6 are identified - I would like to know if plating on different stiffnesses alters the expression of PIEZO1 and HDAC6 either by qPCR or ideally at the protein level

Do the Piezo1 KD, HDAC KD alter cell viability? Live/dead stain would be useful

We thank the reviewer for these suggestions. We have now included Western blot data showing the levels of piezo1 and HDAC6 on 12 and 72 kPa hydrogels.

We have now included Supplementary Figure S1D and Supplementary Figure S5E showing that piezo1 and HDAC6 depletion does not influence VSMC viability. Supplementary Figure 6C shows piezo1 and HDAC6 levels on 12 and 72 kPa hydrogels.

© 2024. Published by The Company of Biologists under the terms of the Creative Commons Attribution License (https://creativecommons.org/licenses/by/4.0/).

To accommodate this new data, the Results section now reads:

Lines 194-195

'Piezo1 depletion had no effect on VSMC viability (Supplementary Figure S1D).'

Lines 293-294

'HDAC6 depletion had no effect on VSMC viability (Supplementary Figure S5E).'

Lines 312-316

'Finally, we performed WB to determine whether piezo1 and HDAC6 levels are influenced by matrix rigidity. As previously reported, piezo1 levels appeared to decrease in VSMCs on rigid hydrogels compared to pliable hydrogels. HDAC6 levels remained similar in VSMCs on pliable and rigid hydrogels (Supplementary Figure S6C).'

Second decision letter

MS ID#: JOCES/2024/262310

MS TITLE: A microtubule stability switch alters isolated vascular smooth muscle calcium flux in response to matrix rigidity.

AUTHORS: Robert T Johnson, Finn Wostear, Reesha Solanki, Oliver Steward, Alice Bradford, Christopher Morris, Stefan Bidula, and Derek T Warren
ARTICLE TYPE: Research Article

I am happy to tell you that your manuscript has been accepted for publication in Journal of Cell Science, pending standard publication integrity checks.

Reviewer 1

Advance summary and potential significance to field

See comments from first review

Comments for the author

Thank you for addressing my comments and adding the additional details.

Reviewer 2

Advance summary and potential significance to field

The authors have addressed all of the points that I initially raised. I have no more comments on the manuscript and recommend that it is now accepted for publication.

Comments for the author

The authors have addressed all of the points that I initially raised. I have no more comments on the manuscript and recommend that it is now accepted for publication.

© 2024. Published by The Company of Biologists under the terms of the Creative Commons Attribution License (https://creativecommons.org/licenses/by/4.0/).

Reviewer 3

Advance summary and potential significance to field

The authors have addressed my concerns well and I congratulate them on the high quality of the revised manuscript.

Comments for the author

The authors have addressed my concerns well and I congratulate them on the high quality of the revised manuscript.

© 2024. Published by The Company of Biologists under the terms of the Creative Commons Attribution License (https://creativecommons.org/licenses/by/4.0/).
